# Iodine-Mediated Alkoxyselenylation of Alkenes and Dienes with Elemental Selenium

**DOI:** 10.3390/molecules27196169

**Published:** 2022-09-20

**Authors:** Evgeny O. Kurkutov, Bagrat A. Shainyan

**Affiliations:** A. E. Favorsky Irkutsk Institute of Chemistry, Siberian Branch, Russian Academy of Sciences, 1 Favorsky Str., 664033 Irkutsk, Russia

**Keywords:** elemental selenium, alkenes, dienes, iodine-mediated reaction, alkoxyselenylation

## Abstract

A one-pot synthesis of linear and cyclic β-alkoxyselenides is developed through the iodine-mediated three-component reaction of elemental selenium with alkenes (dienes) and alcohols. Selenylation of 1,5-hexadiene gives 2,5-di(methoxymethyl)tetrahydroselenophene and 2-methoxy-6-(methoxymethyl)tetrahydro-2*H*-selenopyran via the 5*-exo-trig* and 6-*endo-trig* cyclization. 1,7-Octadiene affords only linear 1:2 adduct with two terminal double bonds. 1,5-Cyclooctadiene results in one diastereomer of 2,6-dialkoxy-9-selenabicyclo [3.3.1]nonanes via 6-*exo-trig* cyclization. With 1,3-diethenyl-1,1,3,3-tetramethyldisiloxane, the first ring-substituted representative of a very rare class of heterocycles, 1,4,2,6-oxaselenadisilinanes, was obtained at a high yield.

## 1. Introduction

Organoselenium compounds are valuable reagents in organic and organoelement synthesis [1,2,3,4] and demonstrate diverse biological activities [5,6,7,8,9]. For example, 4-phenylselenyl-7-chloroquinoline (4-PSQ) exerts acute anti-inflammatory effects on apar with the widely used drug Meloxicam [10]. Some organoselenium compounds affect mood and exhibit high antidepressant activity [11,12]. They possess powerful radical scavenging and broad anti-HIV activity [13] and are capable of blocking the replication of SARS-CoV-2 [14,15,16]. One of the actively studied properties of organoselenium compounds is their glutathione peroxidase activity, [17,18] protecting living organisms against oxidative stress. All of this shows that organoselenium compounds are good candidates for the medicines of the future and promotes the search for new methods of their synthesis and investigation.

**Scheme 1:**
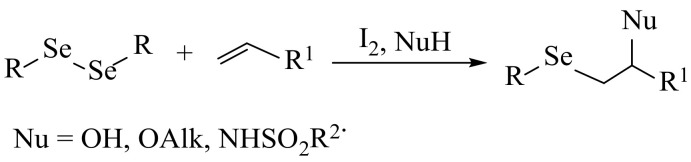
Iodine-mediated selenylation of alkenes with organic diselenides.

Nowadays, the iodine-mediated reactions of alkene functionalization are actively used in organic synthesis [19,20,21] since molecular iodine is non-toxic, inexpensive, safe, and easy to use in the handling of reagents. These reactions are employed in the synthesis of organoselenium compounds, for example, for selenylation of alkenes with organic diselenides via the formation of a Se–C bond, Scheme 1 [22,23,24]. 

The method is promising, although the scope of the substrates was limited to alkenes and did not include other unsaturated substrates. The drawbacks of this method are the impossibility to obtain the Se-containing heterocycles and the use, as a rule, of only diphenyl (or diaryl) diselenides. The main methods of the synthesis of symmetrical difunctionalized and cyclic selenides are based on the reactions of nucleophilic selenium reagents (selenolates and selenols) with electrophiles such as epoxides [25,26,27]. An alternative approach is the electrophilic addition of inorganic selenium halides to alkenes [28,29,30], leading to β-haloselenides (mustard gas analogs).

The drawbacks of such selenium-containing reagents as organic diselenides, organoselenyl halides, inorganic selenium halides, selenols, etc., are their high toxicity, low stability and inability to store for a long time. In contrast, elemental selenium is low-toxic and easy to store, transport and handle and thus has significant advantages as a reagent for the synthesis of organoselenium compounds [4,31].

**Scheme 2:**
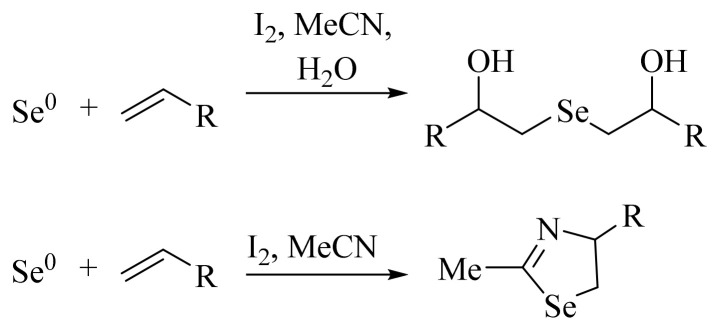
Previous works [32,34].

Recently, we first reported the possibility of introducing a selenium atom into the organic chain by using molecular iodine in the reaction of hydroxyselenylation of alkenes with elemental selenium in an aqueous organic medium, resulting in the formation of bifunctionalized symmetrical β,β-dihydroxyselenides [32]. The reaction with cycloalkenes was shown to proceed stereoselectively as *anti*-addition, resulting exclusively in the *trans*,*trans*-diastereomers [32,33]. In distinction to that, in dry acetonitrile the iodine-mediated reaction of elemental selenium with alkenes proceeded as a Ritter-type reaction with an interception of the solvent (acetonitrile) and resulted in assembling the 1,3-selenazoline ring, Scheme 2 [34]. 

**Figure 1:**
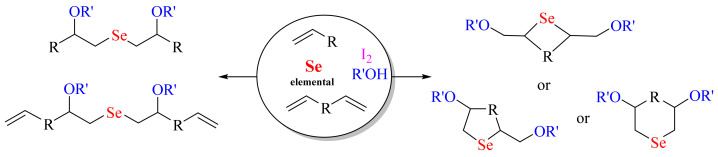
Possible products of alkoxyselenylation of alkenes and dienes.

Here, we report a one-pot procedure for the three-component synthesis of cyclic and linear β,β-alkoxyselenides based on the iodine-mediated alkoxyselenylation of alkenes and dienes with elemental selenium and alcohols. The method takes advantage of using elemental selenium and alcohols as the reagents and allows the selective synthesis of selenium-containing compounds; possible structures are presented in Figure 1. 

The novelty of the present study is not only in extending the reaction of hydroxyselenylation [32] to alkoxyselenylation but also in including, in addition to alkenes, the earlier not-studied dienes as the substrates, which can (and do) result in the formation of novel selenium heterocycles (*vide infra*).

## 2. Results and Discussion

**Figure 2:**
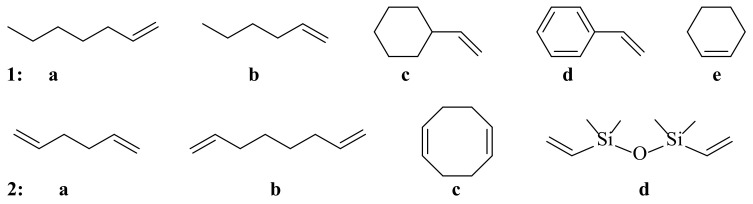
The studied alkenes **1a**–**e** and dienes **2a**–**d**.

The investigated alkenes **1a**–**e** and dienes **2a**–**d** are depicted in Figure 2.

**Scheme 3:**
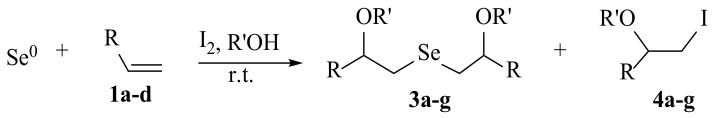
Synthesis of selenides **3a**–**g**.

**Figure 3:**
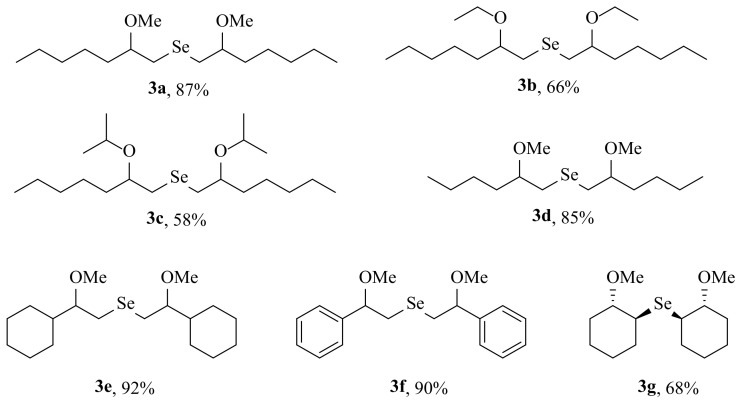
Scope of selenides **3** *. * A total of 3.5 mmol alkene **1a**–**e**, 1 mL of alcohol, 1 mmol of fine-ground Se and I_2_ (2 mmol for **3a**–**c** or 1 mmol for **3d**–**g**), r.t., 40 h.

The reaction of alkenes **1a**–**e** with selenium in alcohols proceeded under mild conditions in one preparative step to give β,β-dialkoxyselenides **3a**–**g** at good yields, taking into account the conversion (Scheme 3, Table 1). The conversion was estimated by subtracting the amount of unreacted (filtered off) selenium from that taken in the reaction. The reaction was highly regioselective; with unsymmetrical alkenes **1a**–**d,** only selenides **3** with the selenium atom at the terminal olefinic carbons were formed. Selenides **3a–f** have two chiral centers which were formed as mixtures of diastereomers in the ratio of ca. 1:1, as proved by the presence of two ^77^Se NMR signals of equal intensity and the doubled sets of signals in the ^13^C NMR spectra. With cyclohexene, only two diastereomers were formed in the ratio of 1:1, although molecule **3g** has four asymmetric centers allowing four diastereomers to be formed. Based on our previous results on the exclusive formation of the *trans*-products of hydroselenylation of cycloalkenes, we assumed stereoselective *anti*-addition and formation of product **3c** as the *d,l* and *meso* diastereomers with *trans*-arrangement of the methoxy groups and the selenium atom. The yields of compounds **3** are given in Figure 3. ^1^H NMR spectroscopy revealed the formation of trace amounts of by-products of iodomethoxylation **4**, which can easily be separated by eluting with CCl_4_. Independently synthesized compounds **4** do not react with selenium under the conditions of the reaction; so, they are not intermediates on the way to the target products **3**.

Optimization of the reaction conditions was performed by varying the solvent, the iodine source and its amount, temperature and reaction time for the reaction of heptene-1 with methanol (Table 1). With 1 or 1.5 equivalents of iodine, the conversion is incomplete (entries 1–4) but increases with time. With two equivalents of iodine, the conversion is complete in 40 h (entry 5) without an increase in the number of by-products **4**. When using five equivalents of methanol (2.5 molar excess), selenide **3a** is formed selectively at a 79% yield (entry 10), but the conversion is rather low. A high conversion of 86% was achieved when using methanol as the solvent (entry 4). The use of catalytic amounts of iodine in combination with DMSO is known [21,22] when iodine is recycled by oxidation of the formed hydrogen iodide with DMSO. However, in our case, the use of DMSO shows low conversion and selectivity affording a mixture of adducts (entry 9). The increase in the temperature to 50 °C lowers the yield (entry 7). Since molecular iodine acts as an oxidant, we tried to replace it with N-iodosuccinimide; indeed, the rate of the reaction increased, but simultaneously increased the number of by-products **4**, up to 30% (NIS, entry 8). No reaction occurs in the absence of iodine or its substitute. To summarize, the most favorable condition is the use of a two-fold excess of iodine in methanol at room temperature. Alkoxyselenylation of alkenes **1a**–**e** was performed under the conditions providing the highest yield of the target selenides (entries 2 and 5) in spite of incomplete conversion, which can be increased by prolonging the reaction time (entry 3).

**Scheme 4:**
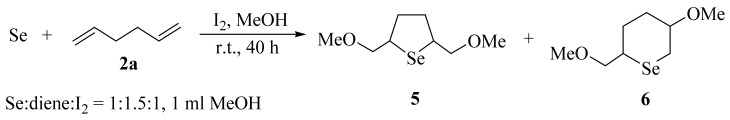
Synthesis of selenides **5** and **6**.

With the results for alkenes **1a**–**e** in mind, one can expect the formation of various products of methoxyselenylation of dienes **2a**–**d** via addition to one or both double bonds of the diene, or the reaction with two diene molecules, or even the products of cyclization or oligomerization. The unpredictability of the reaction is exacerbated by the fact that it is very poorly studied, and the nature of active intermediates is not clear, taking into account that elemental selenium exists as a mixture of single chains, Se_8_ cyclic molecules, or polymer nanoparticles [35] and can form a mixture of iodides of general formula Se_n_I_2_. However, to our surprise and delight, the reaction of methoxyselenylation of 1,5-hexadiene **2a** led selectively to the isomeric products of cyclization, 2,5-di(methoxymethyl)tetrahydroselenophene **5** and its six-membered isomer, 5-methoxy-2-(methoxymethyl)tetrahydro-2*H*-selenopyran **6** in the ratio of 1:2 and an 86% total yield, Scheme 4.

Compounds **5** and **6** are formed as two diastereomers in the ratio of ca. 1:1, as proved by the presence of doubled signals in the ^13^C and ^77^Se NMR spectra of the products. The products could not be completely separated into pure diastereomers, but we succeeded in the isolation of the fractions of compound **6** enriched with diastereomer *1* (80:20) or diastereomer *2* (35:65), which allowed the assignment of the signals to specific diastereomers (see spectra S26–S32 in the Supplementary Materials). 

**Scheme 5:**
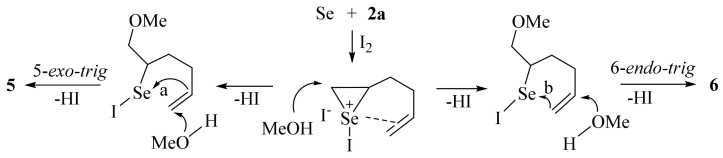
Proposed mechanism of forming heterocycles **5** and **6**.

Note that no linear adducts were formed, even with a large excess of diene **2a**. Remarkably, while the formation of products **3** in Scheme 1 obeys the Markovnikov rule (addition of MeO nucleophile to the internal olefinic carbon), the reaction with 1,5-hexadiene **2a** proceeded as an anti-Markovnikov addition. Apparently, this is due to the effect of the second double bond, which can interact with the selenium atom in the intermediate seleniranium cation with a subsequent opening by methanol via the 5*-exo-trig* or 6-*endo-trig* mode, both of which are favorable according to Baldwin’s rules [36], Scheme 5. 

**Scheme 6:**
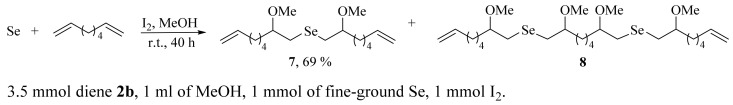
Synthesis of selenide **7**.

In contrast, with a higher homologue of diene **2a** and 1,7-octadiene **2b**, the reaction proceeded with the formation of 1:2 linear adduct, 8,8′-selenobis(7-methoxyoct-1-ene) **7**, with two terminal double bonds being retained, and 2:3 linear adduct, 3,16-dihex-5-en-1-yl-7,12-dimethoxy-2,17-dioxa-5,14-diselenaoctadecane **8**. As with alkenes **1a**–**d**, the addition follows the Markovnikov rule, probably due to a more remote second double bond than in the case of diene **2a**, Scheme 6. 

No cyclic products were detected in the reaction mixture. With a large excess of the diene (Se:**2b** = 1:3), selenide **7** is practically the only product of the reaction. For the equimolar ratio, it still is the main product, but a fraction enriched with compound **8** was also isolated via column chromatography. The structure of **8** was validated through ^1^H NMR spectroscopy and mass spectrometry. The relative intensity of the methylene proton signals with respect to olefinic protons is notably larger than for monoselenide **7**, and the mass spectrum contains a peak with *m*/*z* 614, corresponding to the molecular ion [C_28_H_54_O_4_Se_2_]^+^. 

**Scheme 7:**
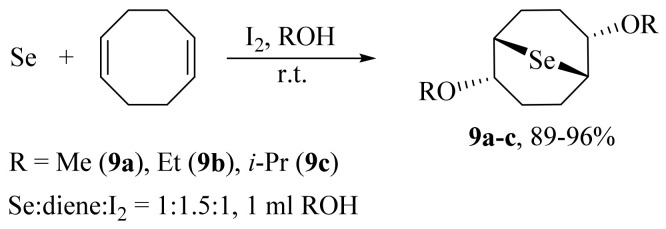
Synthesis of selenide **9**.

For comparison, the reaction with 1,5-cyclooctadiene was investigated. The reaction proceeds regioselectively to give only one diastereomer of 2,6-dialkoxy-9-selenabicyclo [3.3.1]nonanes **9a**–**c**, apparently via alkoxyselenylation at one double bond followed by the 6-*exo-trig* cyclization [36]. Based on the common mechanism of *anti*-addition via the ring opening of the intermediate seleniranium cation and the earlier proved *trans* structure of the products of hydroxyselenylation of cyclohexene [32,33], it is reasonable to assume that the selenium atom in **9** is in the *trans* position to the alkoxy groups, Scheme 7.

**Scheme 8:**
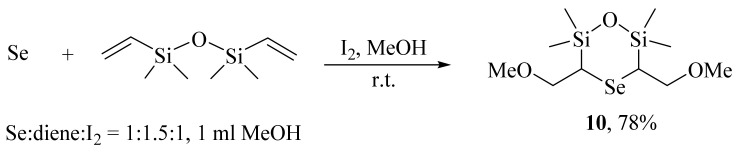
Synthesis of selenide **10**.

Under the same conditions, the iodine-mediated reaction of elemental selenium with 1,3-diethenyl-1,1,3,3-tetramethyldisiloxane and methanol affords 3,5-bis(methoxymethyl)-2,2,6,6-tetramethyl-1,4,2,6-oxaselenadisilinane **10**, Scheme 8.

**Figure 4:**
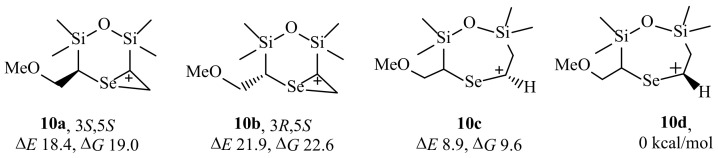
The relative total and free energies of all cations **10** with respect to the most stable cation **10d**.

The structure of compound **10** and, hence, the anti-Markovnikov regioselectivity of addition is proved through the presence of SeCH signals at 22–25 ppm in the ^13^C j-mod NMR spectrum (Figure S47) as well as the value of the ^1^*J*_C-Se_ direct constant of ~58 Hz on the satellites of this signal (Figure S48). Heterocycle **10** is representative of a very rare class of 1,4,2,6-oxaselenadisilinanes and is the first one with substituents in the ring. It is formed of a 2:1 mixture of diastereomers generated by the methanol attack on the *re* or *si* faces of the double bond during the 6-*exo-trig* cyclization. Judging from theoretical calculations, the prevalent diastereomer has both substituents equatorial and is more stable than the *ax-eq* diastereomer by 1.24 kcal/mol in total energy Δ*E* and by 1.52 kcal/mol in free energy Δ*G*. The formula of compound **10** is proved by the presence of a fragment ion with *m*/*z* 297 corresponding to a fragment ion [M + H − MeOH]^+^ formed by protonation and elimination of methanol from **10**. This ion can exist as two diastereomers, each as a carbocation or seleniranium cation. Interestingly, while seleniranium cations **10a^+^** and **10b^+^** are the minima on the MP2 potential energy surface, the six-membered carbocations undergo ring expansion upon geometry optimization to **10c** or **10d**. The relative total and free energies of all cations are given in Figure 4 relative to the most stable seven-membered cyclic cation **10d** [37].

This result raises the question of the manner and relative ability of different substituents to stabilize the adjacent carbocationic center; however, it is beyond the scope of our paper.

**Figure 5:**
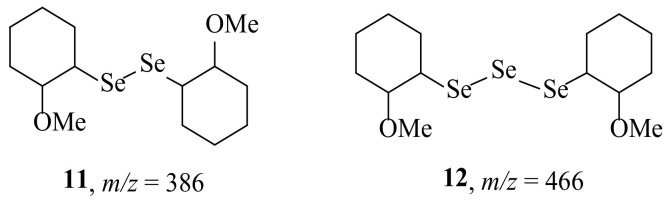
Di- and triselenides **11** and **12**.

Unlike chlorides or bromides, selenium iodides Se_n_I_2_ were not isolated as individuals, but registered in a solution via ^77^Se NMR spectroscopy, the major constituent being Se_2_I_2_ [38]. Based on these data, we assume that the mechanism of hydroxyselenylation is similar to that described in [32], which includes the electrophilic addition of selenium iodides, successive Se–Se bonds rupture via their iodination and, finally, the formation of selenides. This is proved through mass spectrometric detection of di- and triselenides **11** and **12** in the reaction mixture with cyclohexene, as shown in Figure 5.

**Figure 6:**
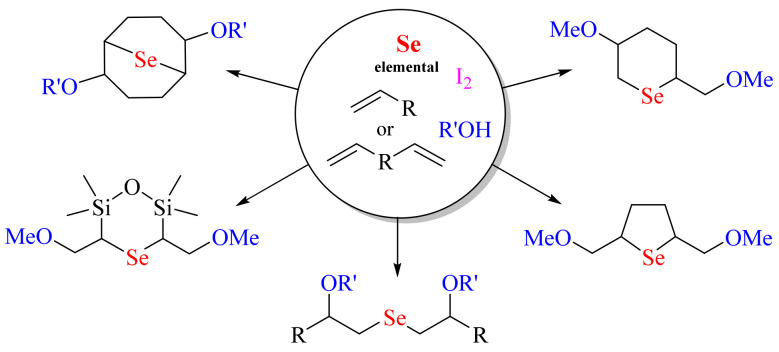
Synthesized linear and cyclic alkoxyselenides.

Therefore, a variety of transformations occur in the system elemental selenium–iodine–alcohol–alkene(diene), opening the way to different selenium-containing linear and heterocyclic compounds, which is illustrated in Figure 6.

## 3. Experimental Section

### 3.1. General Information

Commercially available chemicals and reagents were used without further purification. ^1^H (400.1 MHz), ^13^C (100.6 MHz) and ^77^Se (76.2 MHz) NMR spectra ((Bruker BioSpin GmbH, Rheinstetten, Germany) were recorded on a Bruker DPX-400 spectrometer for 5–10% solutions in CDCl_3_. ^1^H and ^13^C chemical shifts (δ) are reported in parts per million (ppm) relative to the residual solvent peak of CDCl_3_ (δ = 7.27 (^1^H) and 77.10 (^13^C) ppm, respectively). ^77^Se NMR chemical shifts (δ) are given relative to Me_2_Se. High-resolution mass spectra (HRMS) were measured at 70 eV on an Agilent 1200 HPLC chromatograph (Agilent, Santa Clara, CA, USA) with an Agilent 6210 mass spectrometer (HR-TOF-MS, ESI + ionization in acetonitrile, Agilent, Santa Clara, CA, USA). Mass spectra were obtained on a Shimadzu GCMSQP5050A instrument (Shimadzu Corp., Kyoto, Japan). Elemental compositions were determined with a Thermo Scientific Flash 2000 CHNS analyzer (Thermo Fisher Scientific Inc., Milan, Italy). 

### 3.2. General Procedure for the Preparation of Selenides ***3a***–***g*** and ***7***

Alkene **1a**–**e** (or diene **2b**) (3.5 mmol) and alcohol (1 mL) were added to a mixture of fine-ground elemental Se (0.079 g, 1 mmol) and I_2_ (0.508 g, 2 mmol for **3a**–**c** or 1 mmol, 0.254 g for **3d**–**g** and **7**), and the mixture was stirred for 40 h at room temperature. Then, a saturated solution of Na_2_S_2_O_3_ (2 mL) was added, stirred for 10 min, the mixture was diluted with water (10 mL), and Et_2_O (10 mL) was added. The unreacted selenium was filtered off, the water layer extracted with Et_2_O (3 × 10 mL), and the extract was washed with H_2_O (2 × 5 mL), dried with CaCl_2_, and filtered. The solvent was removed in a vacuum to give the crude product, which was purified on silica gel via successive eluting with CCl_4_ and CHCl_3_ to give the corresponding selenide **3a**–**g** and **7**.

### 3.3. General Procedure for the Preparation of Selenides ***5***, ***6***, ***9a***–***c***, and ***10***

Diene **2a**, **2c** or **2d** (1.5 mmol) and alcohol (1 mL) were added to a mixture of fine-ground elemental Se (0.079 g, 1 mmol) and I_2_ (0.254 g, 1 mmol), and the mixture was stirred for 40 h at room temperature. Then, a saturated solution of Na_2_S_2_O_3_ (2 mL) was added, stirred for 10 min, the mixture was diluted with water (5 mL), and Et_2_O (EtOAc for **5**,**6**) (10 mL) was added. The unreacted selenium was filtered off, the water layer extracted with Et_2_O (EtOAc for **5**,**6**) (3 × 10 mL), and the extract was washed with H_2_O (2 × 5 mL), dried with CaCl_2_, and filtered. The solvent was removed in a vacuum to give the crude product, which was purified on silica gel via successive eluting with CCl_4_ and CHCl_3_ to give products **9**, **10**. Isomers **5** and **6** were obtained at an 86% total yield (selenium conversion 79%) as mixtures of regioisomers in the ratio of 2:1. 

### 3.4. Characterization Data of Products ***3a***–***g***, ***5***, ***6***, ***9a***–***c*** and ***10***

2-Methoxy-1-[(2-methoxyheptyl)selanyl]heptane (**3a**):

A yellowish oil, yield 87% (294 mg). ^1^H NMR (CDCl_3_, 400 MHz): δ 3.37 (s, 6H), 3.32−3.29 (m, 2H), 2.77−2.64 (m, 4H), 1.59−1.53 (m, 4H), 1.37−1.27 (m, 12H), 0.89−0.86 (m, 6H). ^13^C NMR (CDCl_3_, 100 MHz): δ 81.45, 56.96, 33.95, 32.00, 28.69, 28.61, 25.07, 22.70, 14.12. ^77^Se NMR (CDCl_3_, 76 MHz): δ 103.46. HRMS, found, 339.1640; calcd for C_16_H_34_O_2_Se [M + H]^+^, 339.1797.

2-Ethoxy-1-[(2-ethoxyheptyl)selanyl]heptane (**3b**).

A yellowish oil, yield 66% (228 mg) (selenium conversion 95%). ^1^H NMR (CDCl_3_, 400 MHz): δ 3.57−3.51 (m, 2H), 3.48−3.40 (m, 2H), 3.40−3.35 (m, 2H), 2.75−2.60 (m, 4H), 1.57−1.52 (m, 4H), 1.48−1.38(m, 12H), 1.36 (t, 6H), 0.87−0.85(m, 6H). ^13^C NMR (CDCl_3_, 100 MHz): δ 79.70, 79.66, 64.65, 34.37, 31.90, 29.21, 29.13, 25.13, 22.61, 15.59, 14.02. ^77^Se NMR (CDCl_3_, 76 MHz): δ 101.76, 100.67. Anal., found: C, 59.52; H, 10.16; Se, 22.14. Calcd for C_18_H_38_O_2_Se: C, 59.16; H, 10.48; Se, 21.61.

2-(Prop-2-yloxy)-1-{[2-(prop-2-yloxy)heptyl]selanyl}heptane (**3c**).

A yellowish oil, yield 58% (211 mg) (selenium conversion 92%). ^1^H NMR (CDCl_3_, 400 MHz): δ 3.69−3.63 (m, 2H), 3.48−3.42 (m, 2H), 2.75−2.61 (m, 4H), 1.64−1.57 (m, 2H), 1.33−1.28(m, 14H), 1.18−1.12 (m, 12H), 0.90−0.87(m, 6H). ^13^C NMR (CDCl_3_, 100 MHz): δ 77.31, 77.24, 70.07, 34.96, 32.01, 30.30, 30.16, 25.30, 23.21, 22.71, 22.66, 14.12. ^77^Se NMR (CDCl_3_, 76 MHz): δ 100.70, 98.29. Anal., found: C, 61.11; H, 10.48; Se, 20.10; calcd for C_20_H_42_O_2_Se: C, 61.04; H, 10.76; Se, 20.07.

2-Methoxy-1-[(2-methoxyhexyl)selanyl]hexane (**3d**):

A yellowish oil, yield 85% (213 mg) (selenium conversion 81%).^1^H NMR (CDCl_3_, 400 MHz): 3.35 (s, 6H), 3.33−3.29 (m, 2H), 2.78−2.65 (m, 4H), 1.61−1.53 (m, 4H), 1.37−1.26 (m, 8H), 0.89 (t, 6H). ^13^C NMR (CDCl_3_, 100 MHz): δ 81.38, 56.89, 33.63, 28.62, 28.54, 27.52, 22.79, 14.05. ^77^Se NMR (CDCl_3_, 76 MHz): δ 97.97, 97.53. Anal. found: C, 53.99; H, 9.48; Se, 25.15. Calcd for C_14_H_30_O_2_Se: C, 54.36; H, 9.77; Se, 25.52.

1,1′-[Selanylbis(1-methoxyethane-2,1-diyl)]dicyclohexane (**3e**):

A yellowish oil, yield 92% (233 mg) (selenium conversion 70%). ^1^H NMR (CDCl_3_, 400 MHz): 3.40 (s, 6H), 3.14−3.10 (m, 2H), 2.77−2.74 (m, 4H), 1.81−1.61 (m, 12H), 1.27−1.00 (m, 10H). ^13^C NMR (CDCl_3_, 100 MHz): δ 86.06, 86.04, 58.24,29.06, 28.23, 26.61, 26.34, 26.30, 26.27. ^77^Se NMR (CDCl_3_, 76 MHz): δ 102.38, 101.77. HRMS, found, 363.1645; calcd for C_18_H_34_O_2_Se [M + H]^+^, 363.1797.

1,1′-[Selanylbis(1-methoxyethane-2,1-diyl)]dibenzene (**3f**):

A yellowish oil, yield 90% (201 mg) (selenium conversion 64%). ^1^H NMR (CDCl_3_, 400 MHz): δ 7.33−7.35 (m, 10H), 4.29−4.23 (m, 2H), 3.22−4.20 (m, 6H), 2.93−2.86 (m, 2H), 2.69−2.64 (m, 2H). ^13^C NMR (CDCl_3_, 100 MHz): δ 141.35, 128.53, 128.01, 126.80, 126.74, 84.55, 56.96, 56.93, 32.12, 31.96. ^77^Se NMR (CDCl_3_, 76 MHz): δ 146.62, 146.12. Anal., found: C, 61.43; H, 5.87, Se, 22.92; calcd for C_18_H_22_O_2_Se: C, 61.89; H, 6.35, Se, 22.60.

1,1′-Selanylbis(2-methoxycyclohexane) **3g**.

A yellowish oil, yield 68% yield (201 mg) (selenium conversion 79%). ^1^H NMR (CDCl_3_, 400 MHz): δ 3.37 (s, 6H), 3.22−3.09 (m, 4H), 2.17−2.01 (m, 4H), 1.71−1.50 (m, 6H), 1.34−1.26 (m, 6H). ^13^C NMR (CDCl_3_, 100 MHz): δ 83.69, 83.50, 56.58, 56.45, 31.98, 31.75, 30.01, 25.60, 25.40, 23.30, 23.15. ^77^Se NMR (CDCl_3_, 76 MHz): δ 327.32. HRMS, found, 307.1178; calcd for C_14_H_26_O_2_Se [M + H]^+^, 307.1171.

2,5-Bis(methoxymethyl)tetrahydroselenophene **5**:

^1^H NMR (CDCl_3_, 400 MHz): δ 3.79−3.75 (m, 2H), 3.62−3.57 (m, 2H), 3.37 (s, 6H), 2.18−2.06 (m, 2H), 1.99−1.72 (m, 2H). ^13^C NMR (CDCl_3_, 100 MHz): δ 77.34, 58.78, 43.14, 43.00, 34.04, 33.67. ^77^Se NMR (CDCl_3_, 76 MHz): δ 312.40, 311.12. GS-MS: *m*/*z* (rel. int., %) 224 [M]^+·^(40), 192 (21), 160 (30), 147 (100). 

5-Methoxy-2-(methoxymethyl)tetrahydro-2*H*-selenopyran (**6**)**,** diastereomer 1: 

^1^H NMR (CDCl_3_, 400 MHz): δ 3.50−3.36 (m, 3H), 3.37 (s, 3H), 3.34 (s, 3H), 3.21−3.14 (m, 1H), 2.83−2.79 (m, 1H), 2.61−2.56 (m, 1H), 2.40−2.34 (m, 1H), 2.11−2.06 (m, 1H), 1.77 −1.66 (m, 1H), 1.32−1.22 (m, 1H). ^13^C NMR (CDCl_3_, 100 MHz): δ 78.88, 76.06, 58.89, 55.94, 35.79, 32.64, 32.52, 22.44. ^77^Se NMR (CDCl_3_, 76 MHz): δ 201.52. GS-MS: *m*/*z* (rel. int., %) 224 [M]^+·^(27), 192 (5), 179 (8), 147 (100). 

5-Methoxy-2-(methoxymethyl)tetrahydro-2*H*-selenopyran (**6**), diastereomer 2:

^1^H NMR (CDCl_3_, 400 MHz): δ 3.62−3.58 (m, 3H), 3.38 (s, 3H), 3.37 (s, 3H), 3.09−3.02 (m, 1H), 2.81−2.76 (m, 1H), 2.71−2.66 (m, 1H), 2.32−2.24 (m, 1H), 2.03−1.95 (m, 1H), 1.75−1.69 (m, 2H). ^13^C NMR (CDCl_3_, 100 MHz): δ 76.18, 75.01, 58.94, 55.84, 32.14, 28.52, 28.14, 20.87. ^77^Se NMR (CDCl_3_, 76 MHz): δ 165.06.

7-Methoxy-8-[(2-methoxyoct-7-en-1-yl)selanyl]oct-1-ene (**7**):

A light yellow oil, yield 69% yield (158 mg) (selenium conversion 63%). ^1^H NMR (CDCl_3_, 400 MHz): δ 5.86−5.76 (m, 2H), 5.03−4.93 (m, 4H), 3.37−3.32 (m, 8H), 2.79−2.66 (m, 4H), 2.09−2.04 (m, 4H), 1.61−1.58 (m, 4H), 1.44−1.38 (m, 4H). ^13^C NMR (CDCl_3_, 100 MHz): δ 138.92, 114.45, 81.33, 56.97, 33.79, 33.75, 29.03, 28.62, 28.55, 24.85, 23.15 ^77^Se NMR (CDCl_3_, 76 MHz): δ 97.94, 97,64. GS-MS: *m*/*z* (rel. int., %) 362 [M]^+·^(1), 221 (14), 189 (21), 95 (100). Anal., found: C, 59.82; H, 9.48, Se, 21.85; calcd for C_18_H_34_O_2_Se: C, 60.19; H, 9.55, Se, 22.30.

2,6-Dimethoxy-9-selenabicyclo [3.3.1]nonane (**9a**):

Colorless oil, yield 96% (141 mg) (selenium conversion 59%). ^1^H NMR (CDCl_3_, 400 MHz): δ 3.89−3.83 (m, 2H), 3.33 (s, 6H), 3.00−3.99 (m, 2H), 2.62−2.57 (m, 2H), 2.20−2.11 (m, 2H), 2.03−1.96 (m, 2H), 1.81−1.70 (m, 2H). ^13^C NMR (CDCl_3_, 100 MHz): δ 81.00, 55.82, 28.85, 28.02, 27.85. ^77^Se NMR (CDCl_3_, 76MHz): δ 287.69. GS-MS: *m*/*z* (rel. int., %) 250 [M]^+·^(37), 218 (10), 179 (24), 71 (100). Anal., found: C, 48.60; H, 7.25, Se, 31.87; calcd for C_10_H_18_O_2_Se: C, 48.20; H, 7.28, Se, 31.68.

2,6-Dimethoxy-9-selenabicyclo [3.3.1]nonane (**9b**):

Colorless oil, yield 91% (189 mg) (selenium conversion 75%). ^1^H NMR (CDCl_3_, 400 MHz): δ 3.97−3.92 (m, 2H), 3.60−3.52 (m, 2H), 3.46−3.39 (m, 2H), 2.95−2.94 (m, 2H), 2.65−2.59 (m, 2H), 2.19−2.09 (m, 2H), 1.98−1.91 (m, 2H), 1.17−1.13 (m, 6H). ^13^C NMR (CDCl_3_, 100 MHz): δ 79.34, 63.39, 29.31, 28.03, 15.74. ^77^Se NMR (CDCl_3_, 76MHz): δ 287.80. Anal., found: C, 51.77; H, 7.89, Se, 28.54; calcd for C_12_H_22_O_2_Se: C, 51.98; H, 8.00, Se, 28.48.

2,6-Bis(propan-2-yloxy)-9-selenabicyclo [3.3.1]nonane (**9c**):

Colorless oil, yield 89% (196 mg) (selenium conversion 72%). ^1^H NMR (CDCl_3_, 400 MHz): δ 4.04−3.99 (m, 2H), 3.73−3.66 (m, 2H), 3.88−3.85 (m, 2H), 2.71−2.66 (m, 2H), 2.20 −2.11 (m, 2H), 1.90−1.82 (m, 2H), 1.15−1.11 (m, 12H). ^13^C NMR (CDCl_3_, 100 MHz): δ 77.18, 68.92, 30.04, 29.64, 28.33, 23.57, 22.46. ^77^Se NMR (CDCl_3_, 76MHz): δ 291.20. Anal., found: C, 55.06; H, 8.58, Se, 25.90; calcd for C_14_H_26_O_2_Se: C, 55.07; H, 8.58, Se, 25.86.

3,5-Dimethoxy-2,2,6,6-tetramethyl-1,4,2,6-oxaselenadisilinane (**10**):

A light yellow oil, yield 78% (140 mg) (selenium conversion 60%). ^1^H NMR (CDCl_3_, 400 MHz): δ 3.71−3.52 (m, 4H), 3.34 (s, 3H), 3.33 (s, 3H), 2.39−2.33 (m, 2H), 0.21−0.19 (m, 12H). ^13^C NMR (CDCl_3_, 100 MHz): δ 74.15, 73.31, 58.54, 25.30, 22.23, 0.75, 0.62, −1.73, −2.29. ^77^Se NMR (CDCl_3_, 76 MHz): δ 160.43, 134.88. ^29^Si NMR (CDCl_3_, 79.5 MHz): δ 5.71, 5.46. HRMS, found, 297.0240; calcd for C_9_H_21_O_2_SeSi_2_ [M + H − MeOH]^+^, 297.0240. Anal., found: C, 36.77; H, 7.22, Se, 31.87; calcd for C_10_H_24_O_3_SeSi_2_: C, 36.68; H, 7.39.

## 4. Conclusions

To summarize, a one-pot procedure for the synthesis of linear and cyclic alkoxy-functionalized organoselenium compounds is proposed, based on the iodine-mediated reaction of elemental selenium with alkenes and dienes in alcohols.

The method has significant advantages over the existing procedures, such as the use of simple and low-toxic reagents, mild conditions, and a diversity of synthesized products.

## Figures and Tables

**Table 1:** Optimization of reaction conditions *.

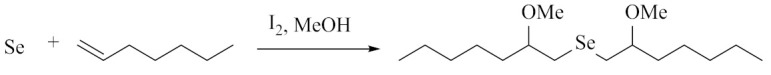						
Entry	Iodine Source (Equiv)	Solvent (mL)	Time, h	*T*, °C	Yield, % **	Se Conversion, %
1	1.0 I_2_	MeCN:MeOH (1:0.1)	40	25	71	54
2	1.0 I_2_	MeOH (1)	40	25	90	68
3	1.0 I_2_	MeOH (1)	120	25	97	88
4	1.5 I_2_	MeOH (1)	40	25	85	86
5	2.0 I_2_	MeOH (1)	40	25	87	~100
6	2.0 I_2_	MeOH (1)	20	25	88	69
7	1.0 I_2_	MeOH (1)	10	50	70	48
8	2.0 NIS	MeOH (1)	20	25	70	63
9	0.2 I_2_	DMSO:MeOH (0.15:0.1)	20	25	Mixture of adducts	27
10	2.0 I_2_	MeOH (0.2) (5 equiv)	40	25	79	57
11	–	MeOH (1)	40	25	0	0

* Reaction conditions: elemental Se (1 mmol), heptene-1 (3.5 mmol) and stirring in MeOH or MeCN:MeOH at 25–50 °C for 10–120 h. ** Iodides **4** formed in the amounts from traces to ca. 10% were not isolated and proved by NMR spectroscopy and mass spectrometry.

## Data Availability

Not applicable.

## Supplementary Materials

The following supporting information can be downloaded at: https://www.mdpi.com/article/10.3390/molecules27196169/s1. Copies of the ^1^H NMR, ^13^C NMR, and ^77^Se spectra for compounds **3a**–**g**, **5**, **6**, **9a**–**c** and **10** can be found in Supplementary Materials.

## Sample Availability

Not available.
