# Supplementary material for: Iodine-Mediated Alkoxyselenylation of Alkenes and Dienes with Elemental Selenium
Source: Molecules. 2022 Sep 20;27(19):6169. doi: 10.3390/molecules27196169 (PMC9572842; doi:10.3390/molecules27196169)
Supplement: Supplementary file 1 [file molecules-27-06169-s001.zip › molecules-1902416-supplementary.pdf]

Supporting Information
for

Iodine-mediated Alkoxyselenenylation of Alkenes and Dienes with Elemental Selenium

Evgeny O. Kurkutov and Bagrat A. Shainyan*

A. E. Favorsky Irkutsk Institute of Chemistry, Siberian Division of the Russian Academy of Sciences, 1 Favorsky Street, 664033 Irkutsk, Russian Federation

* *Correspondence: bagrat@irioch.irk.ru*

Supporting Information

Page

1. General Remarks **S2**

2. Copies of ^{1}H , ^{13}C NMR and ^{77}Se NMR spectra **S3**

General Remarks: ^{1}H (400.1 MHz), ^{13}C (100.6 MHz), and ^{77}Se (76.2 MHz) NMR spectra were recorded on a Bruker DPX-400 spectrometer for 5–10% solutions in $CDCl_3$. ^{1}H and ^{13}C chemical shifts (δ) are reported in parts per million (ppm) relative to the residual solvent peak of $CDCl_3$ (δ = 7.27 (^{1}H) and 77.10 (^{13}C) ppm, respectively). ^{77}Se NMR chemical shifts (δ) are given relative to Me_2Se. High-resolution mass spectra (HRMS) were measured at 70 eV on an Agilent 1200 HPLC chromatograph with Agilent 6210 mass spectrometer (HR-TOF-MS, ESI + ionization in acetonitrile). Mass spectra were obtained on a Shimadzu GCMSQP5050A instrument. Elemental compositions were determined with a Thermo Scientific Flash 2000 CHNS analyzer.

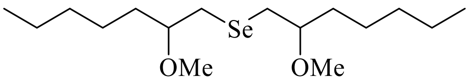

Figure S1. ^{1}H NMR spectrum of compound **3a**

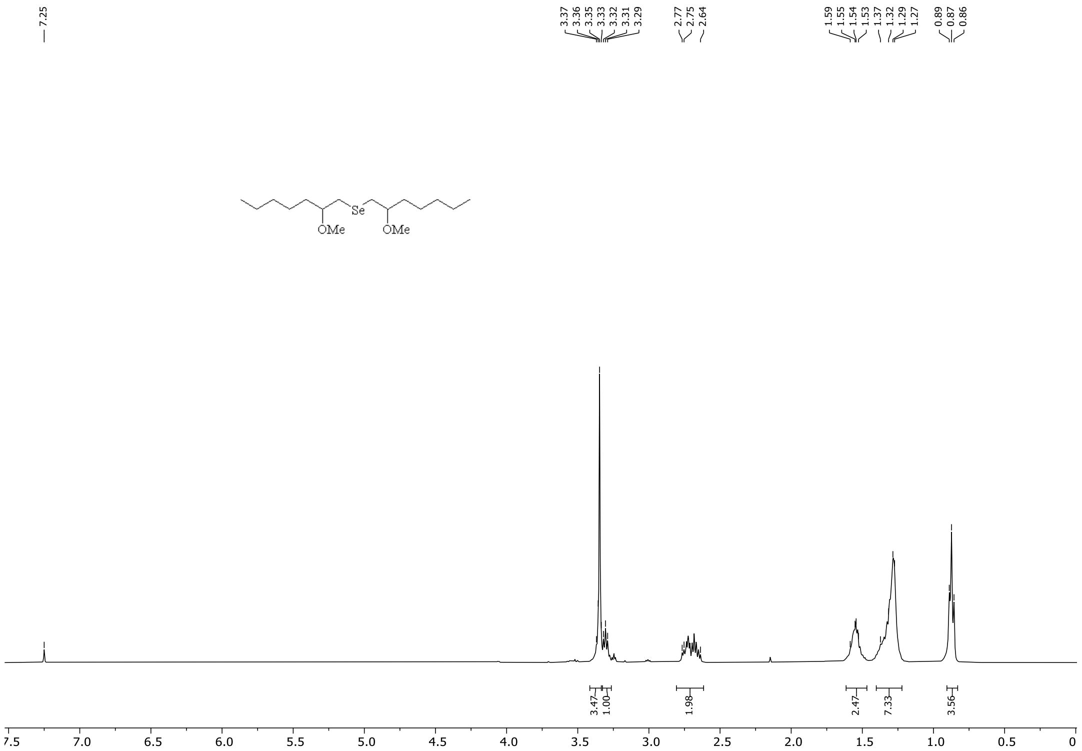

Figure S2. \ ^{13}C NMR spectrum of compound **3a**

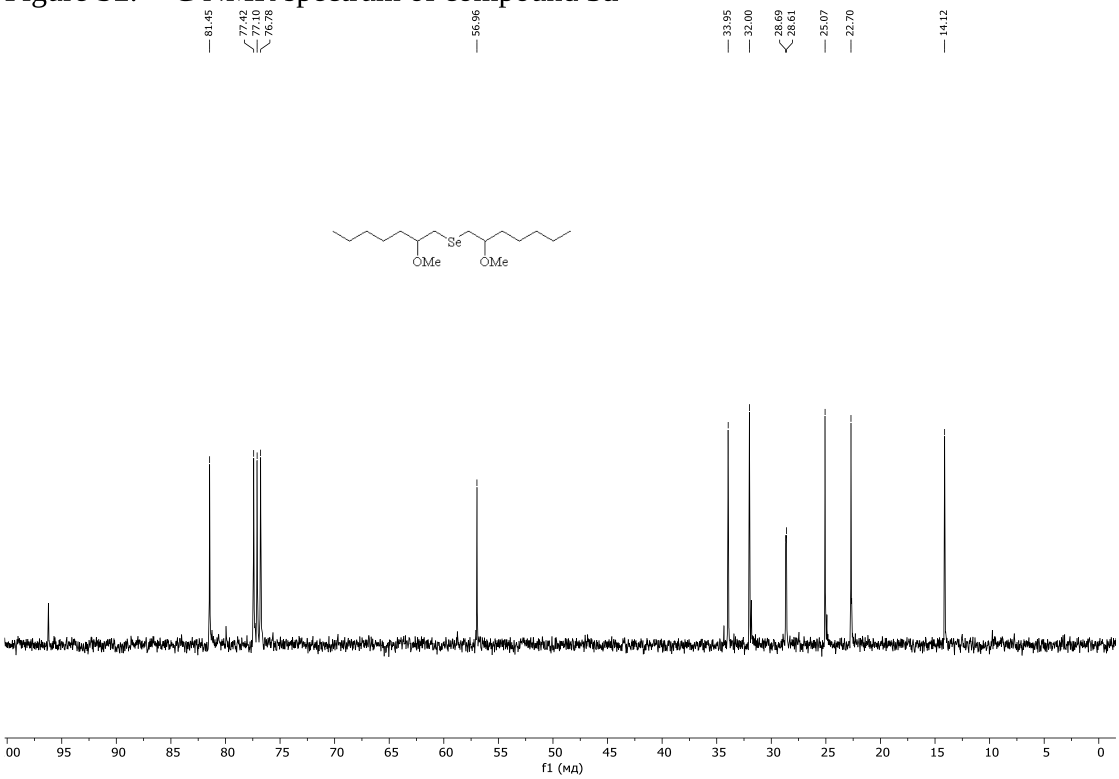

Figure S3. ^{77}Se NMR spectrum of compound **3a**

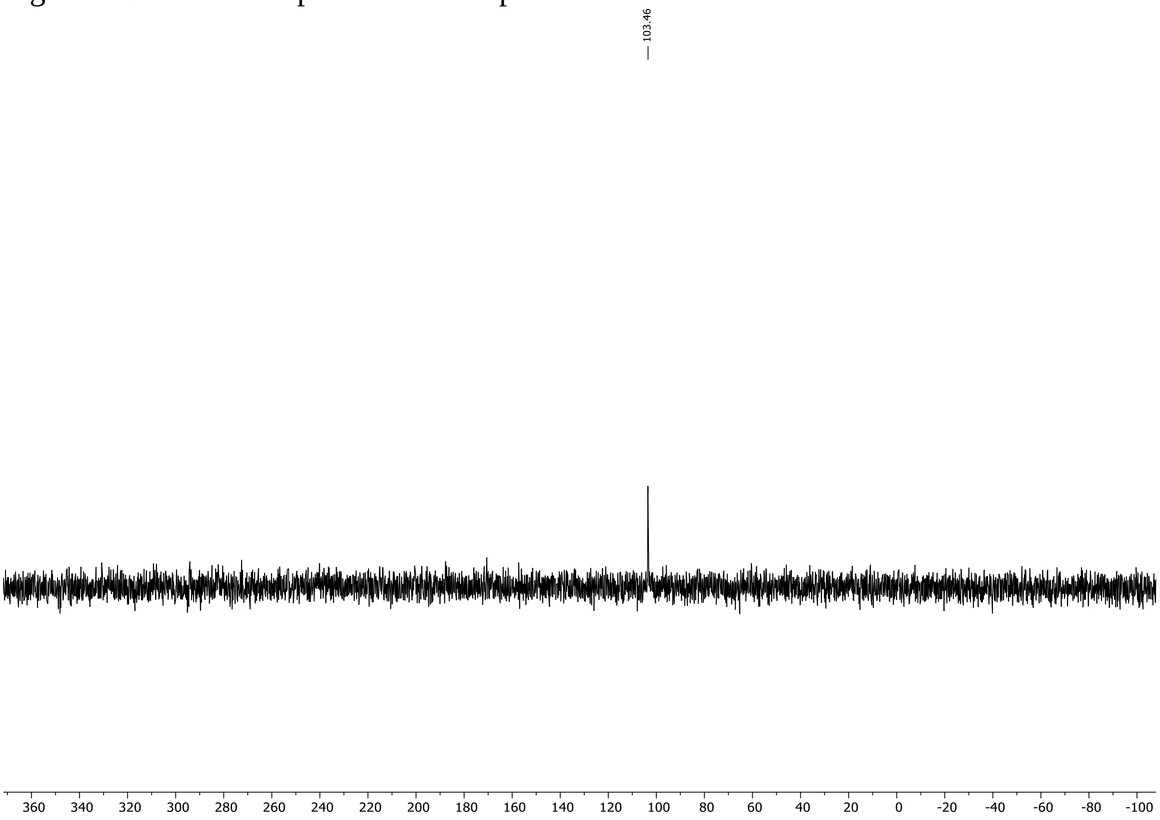

Figure S4. HRMS spectrum of compound **3a**

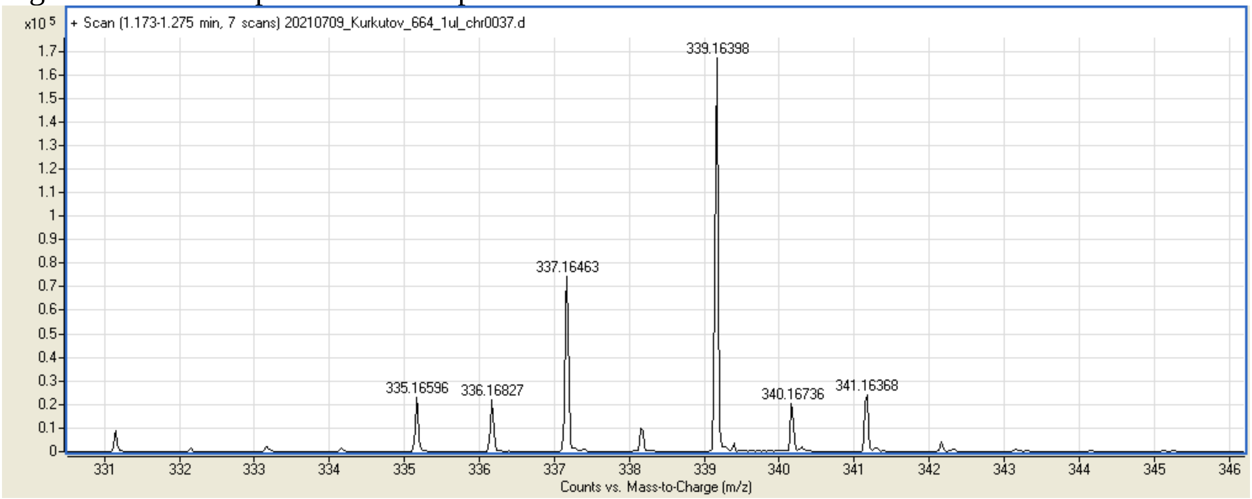

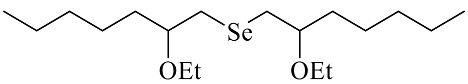

Figure S5. ^{1}H NMR spectrum of compound **3b**

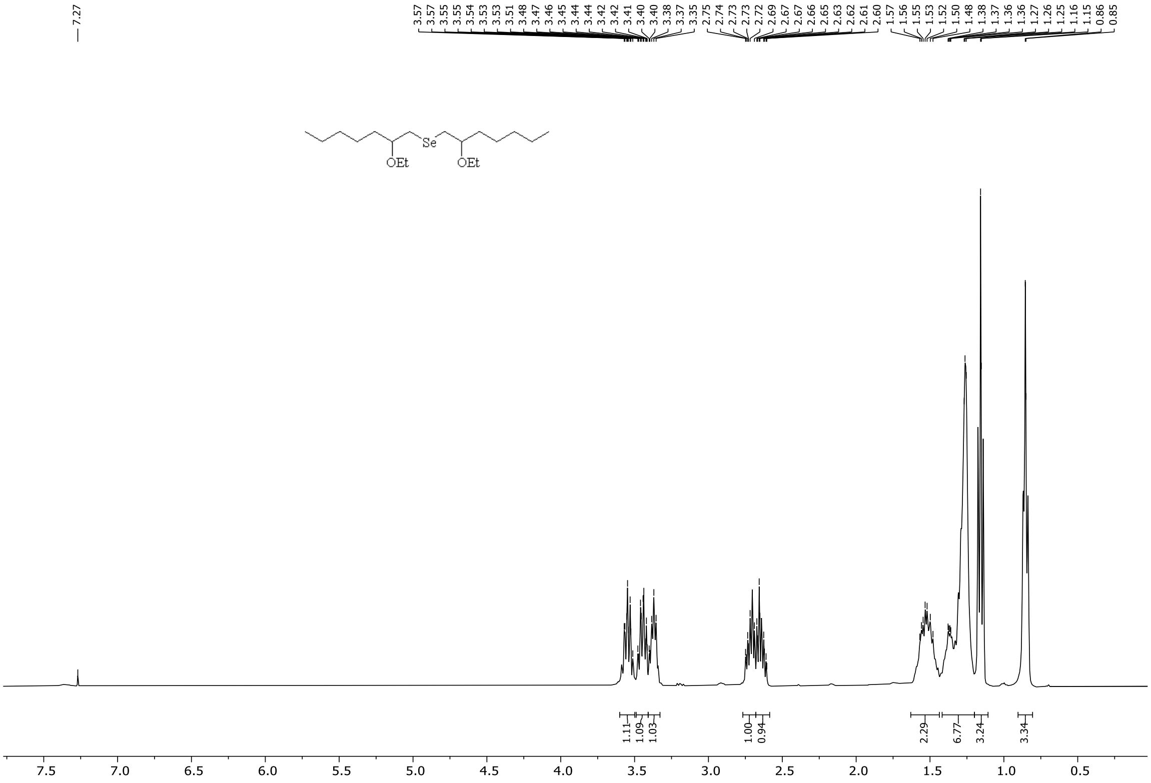

Figure S6. ^{13}C NMR spectrum of compound **3b**

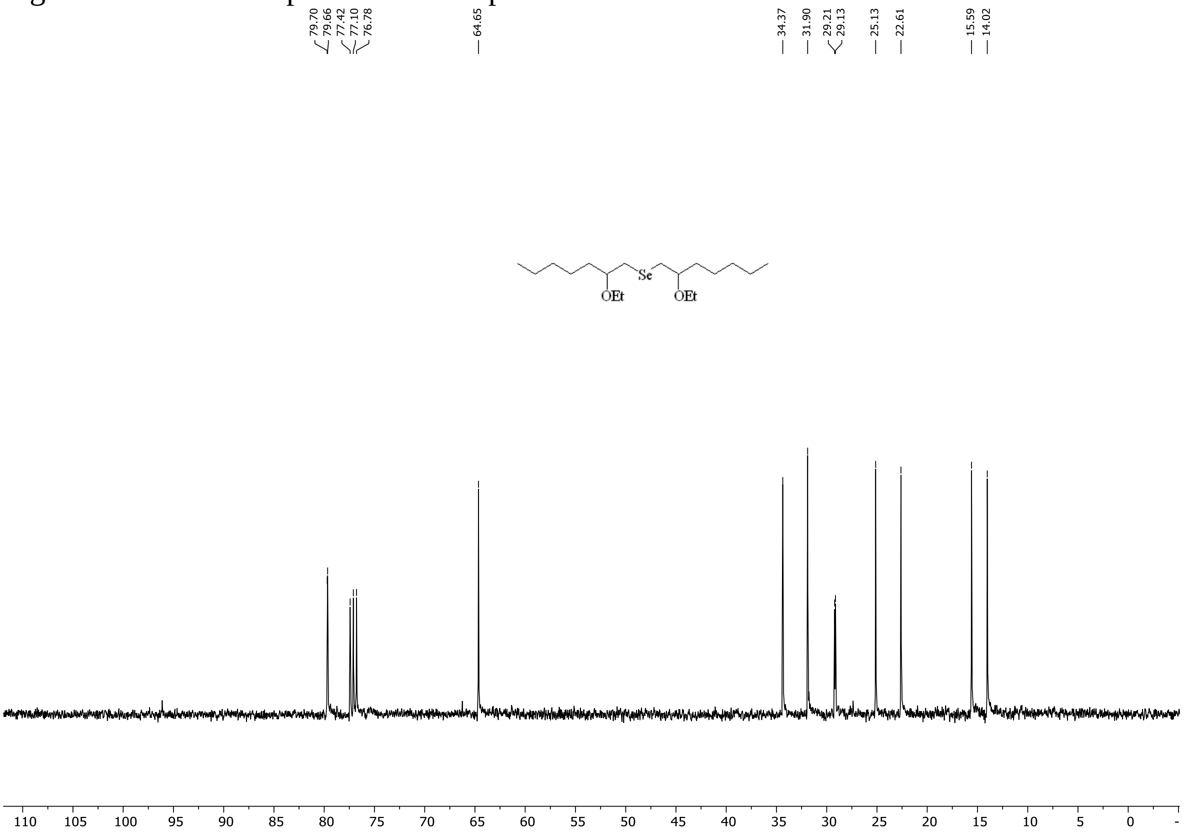

Figure S7. ^{77}Se NMR spectrum of compound **3b**

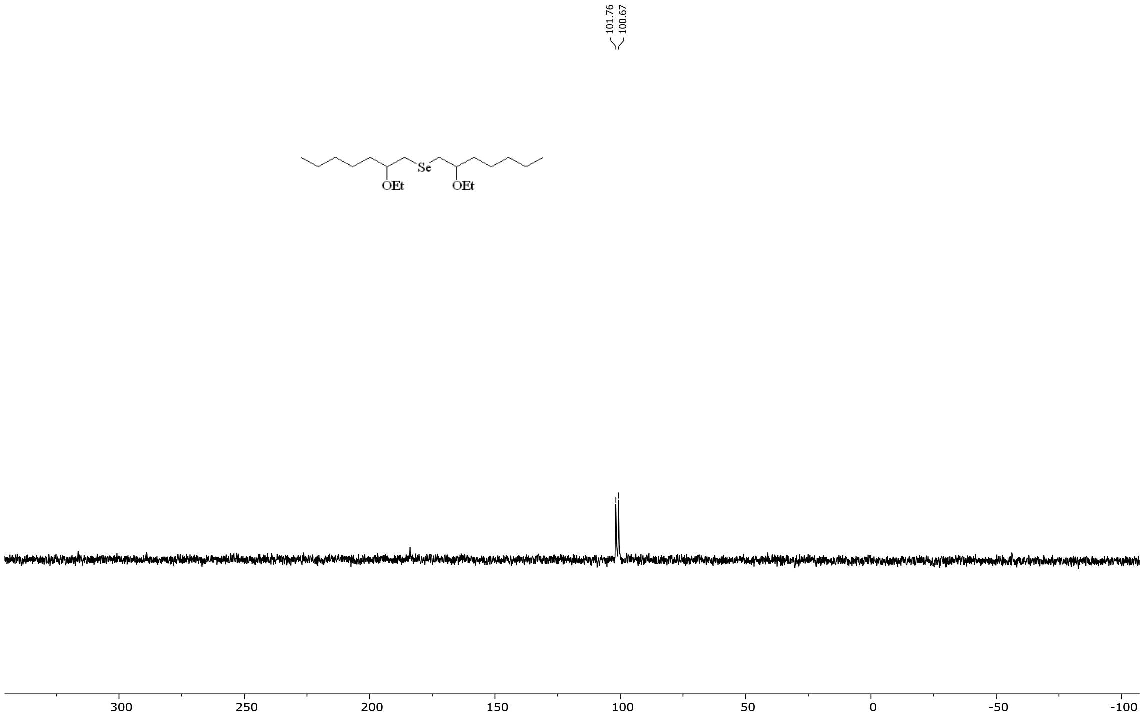

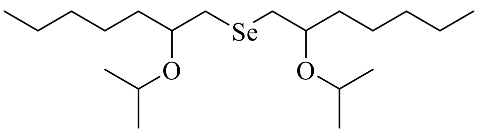

Figure S8. ^{1}HNMR spectrum of compound **3c**

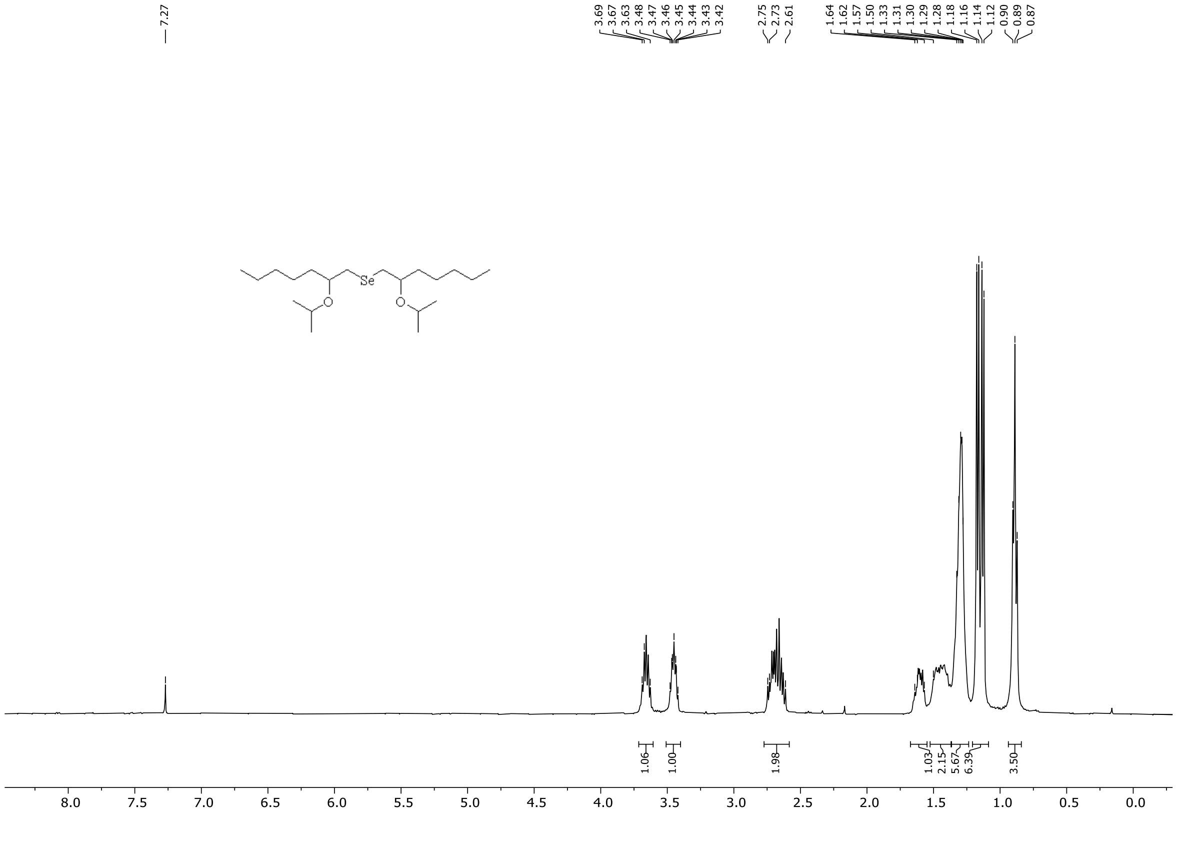

Figure S9. ^{13}C NMR spectrum of compound **3c**

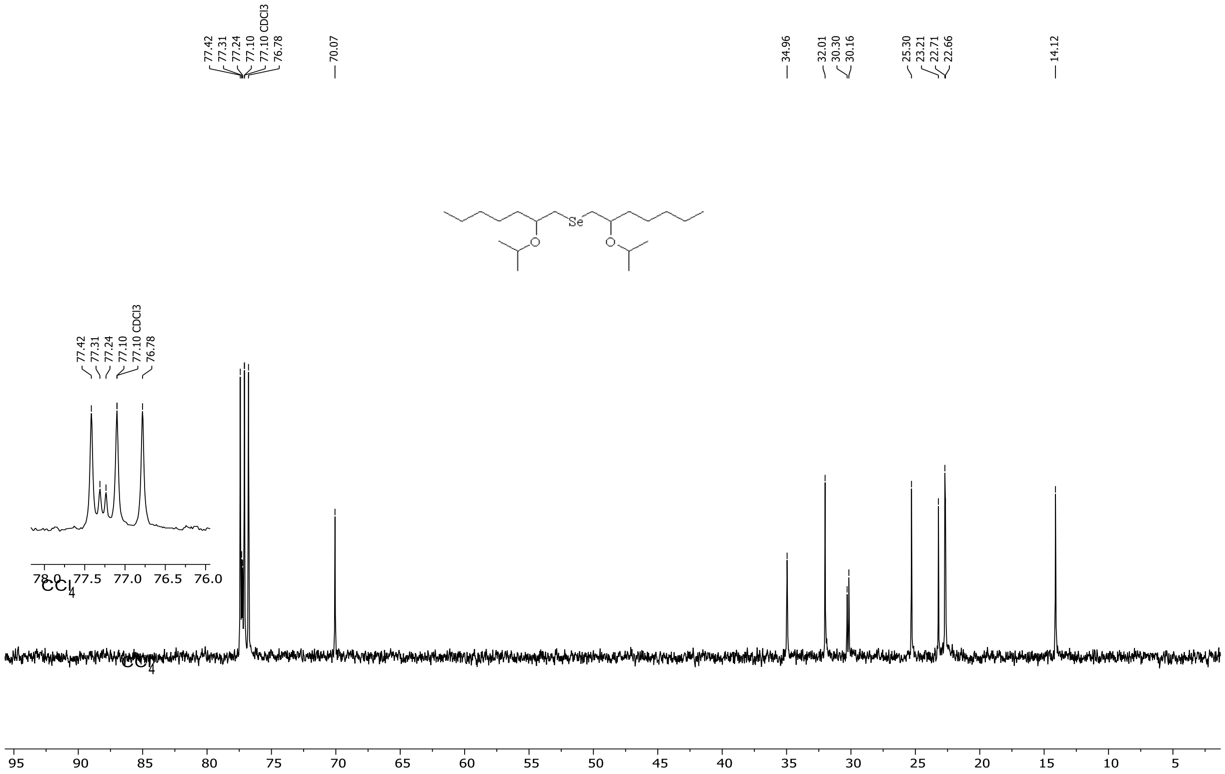

Figure S10. ^{77}C NMR spectrum of compound **3c**

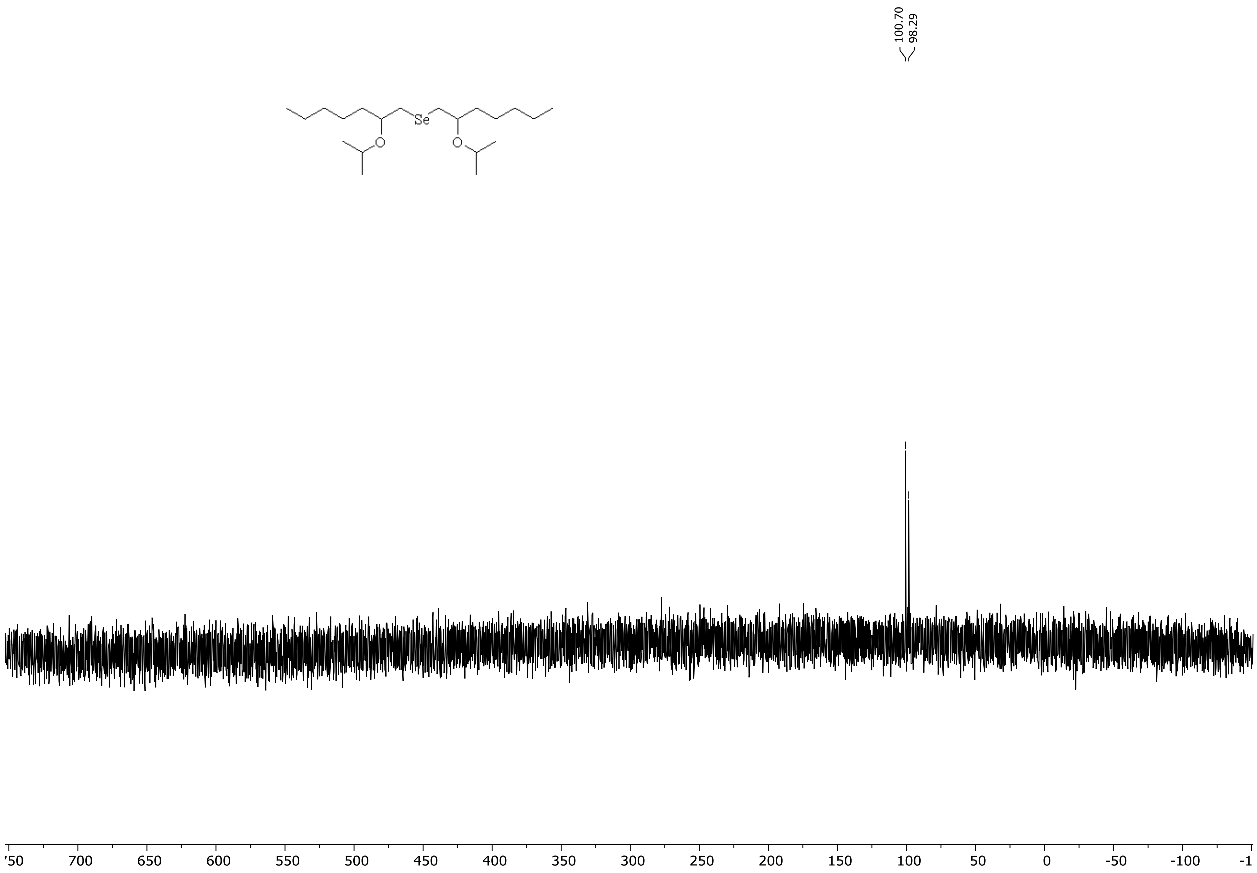

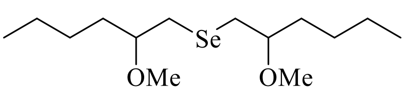

Figure S11. ^{1}H NMR spectrum of compound **3d**

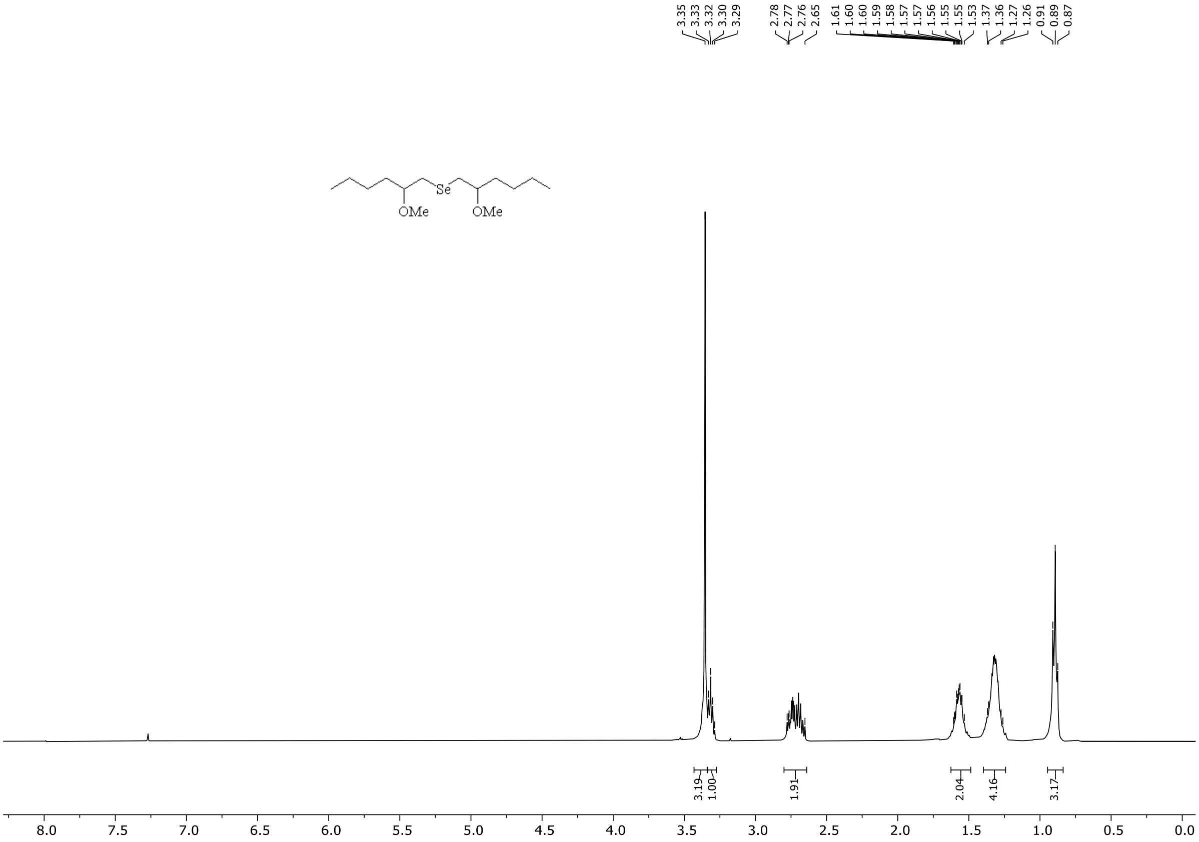

Figure S12. ^{13}C NMR spectrum of compound **3d**

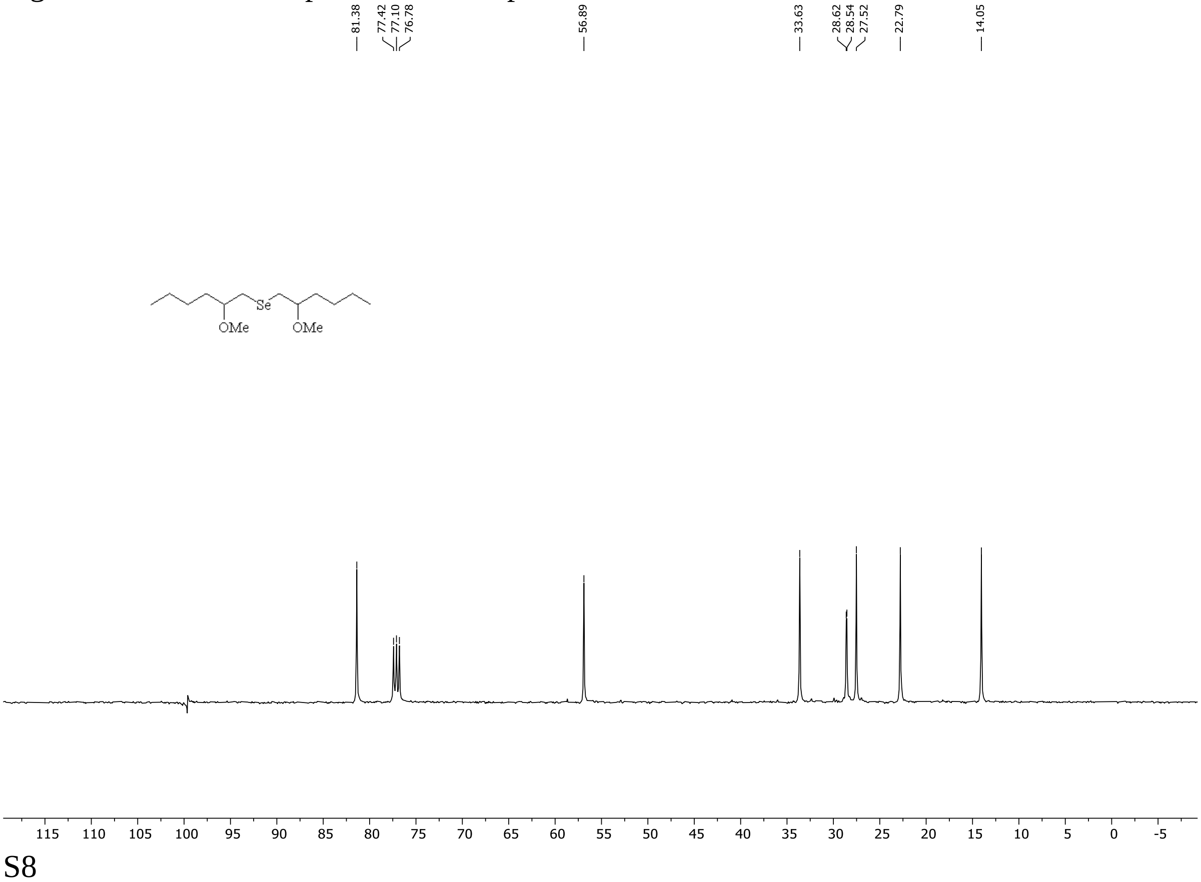

Figure S13. ^{77}Se NMR spectrum of compound **3d**

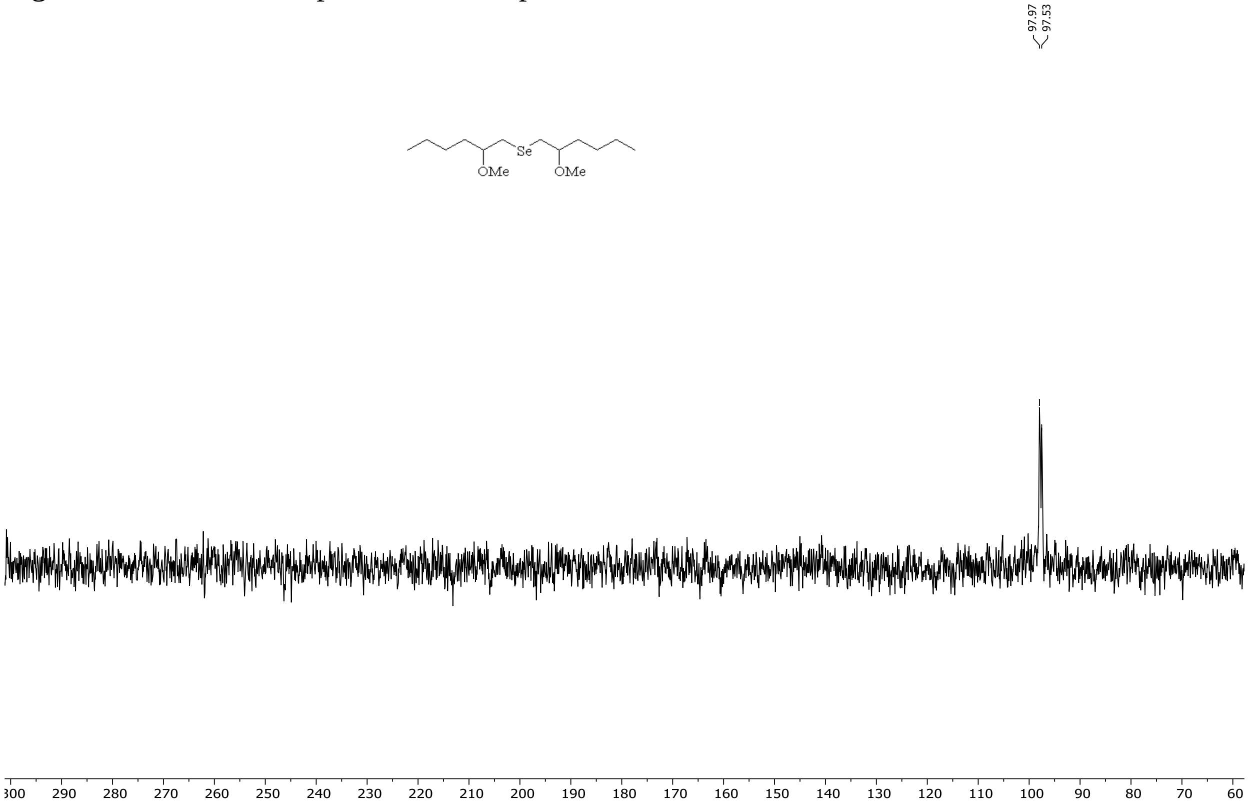

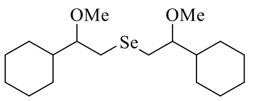

Figure S14. ^{1}H NMR spectrum of compound **3e**

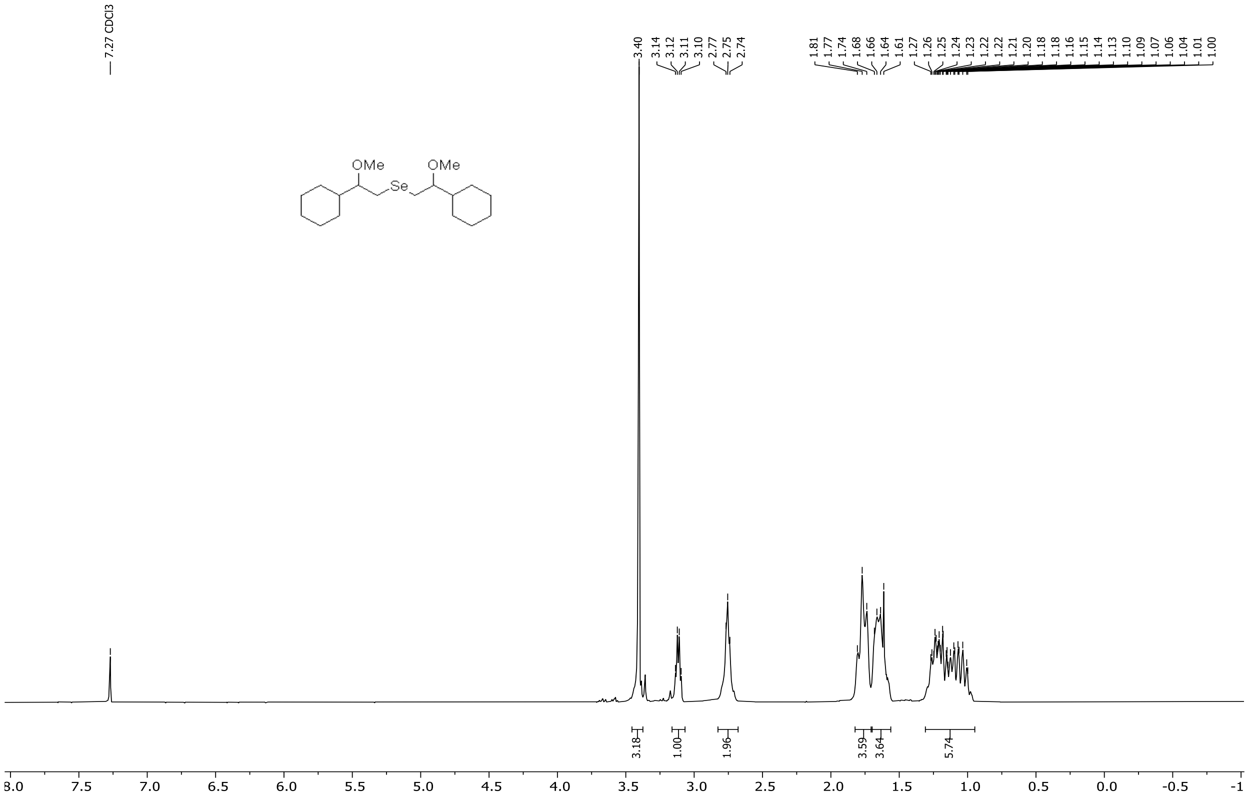

Figure S15. ^{13}C NMR spectrum of compound **3e**

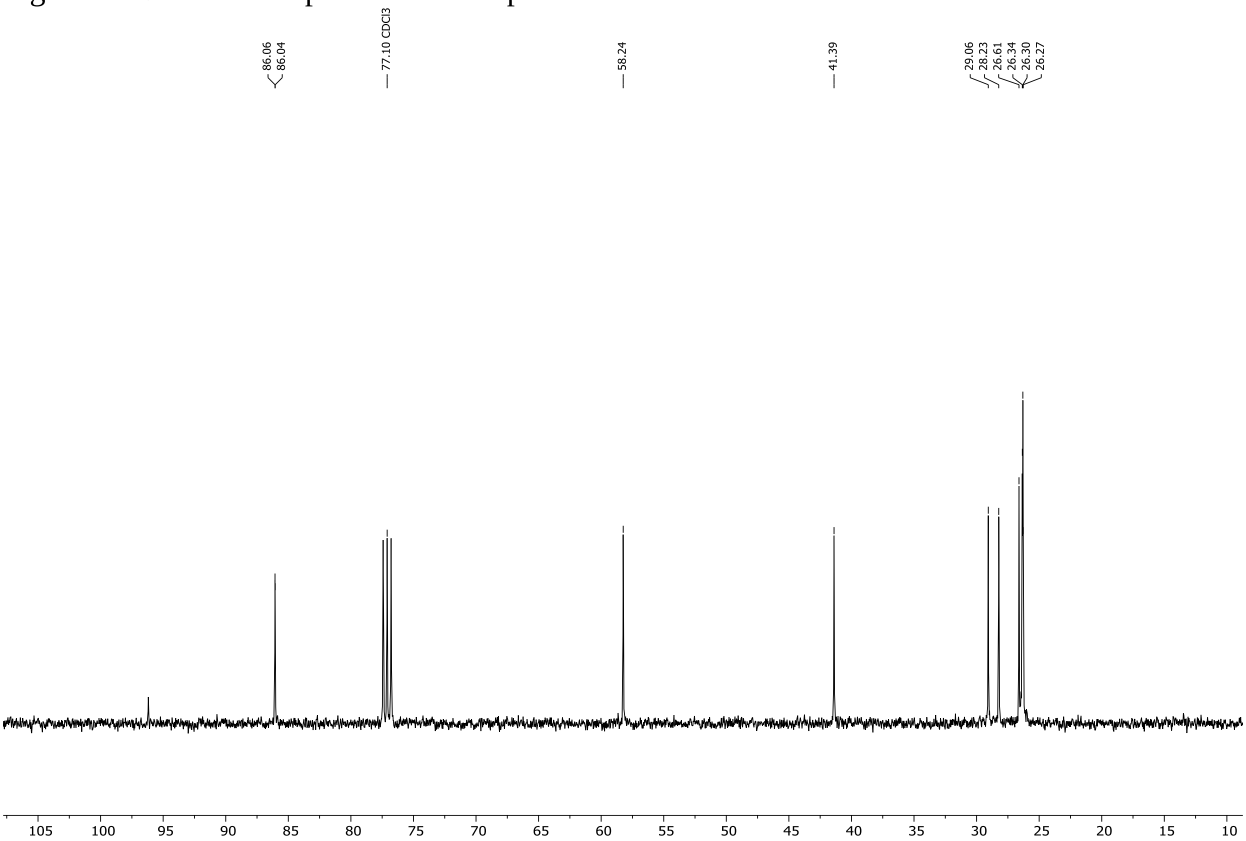

Figure S16. ^{77}Se NMR spectrum of compound **3e**

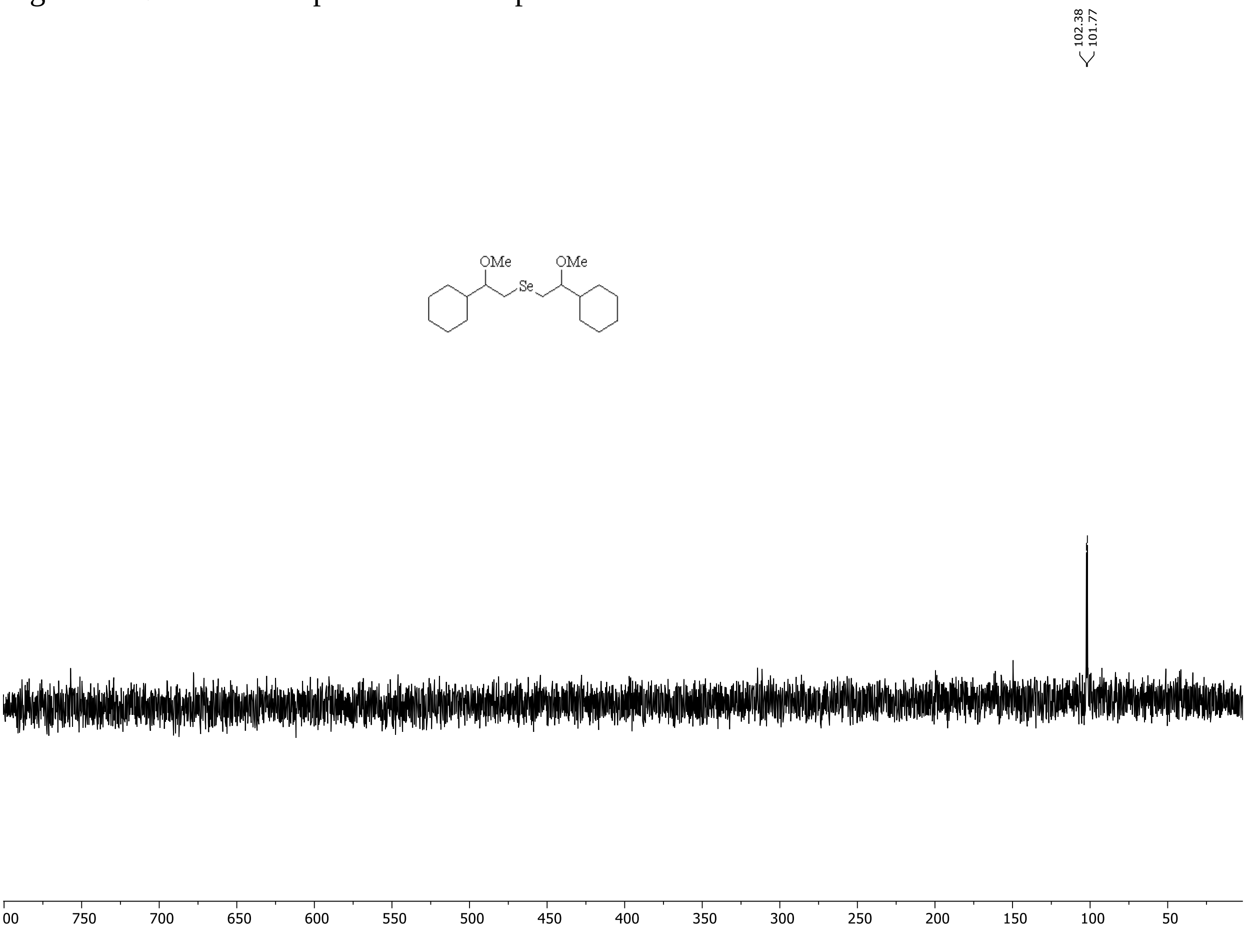

Figure S17. HRMS spectrum of compound **3e**

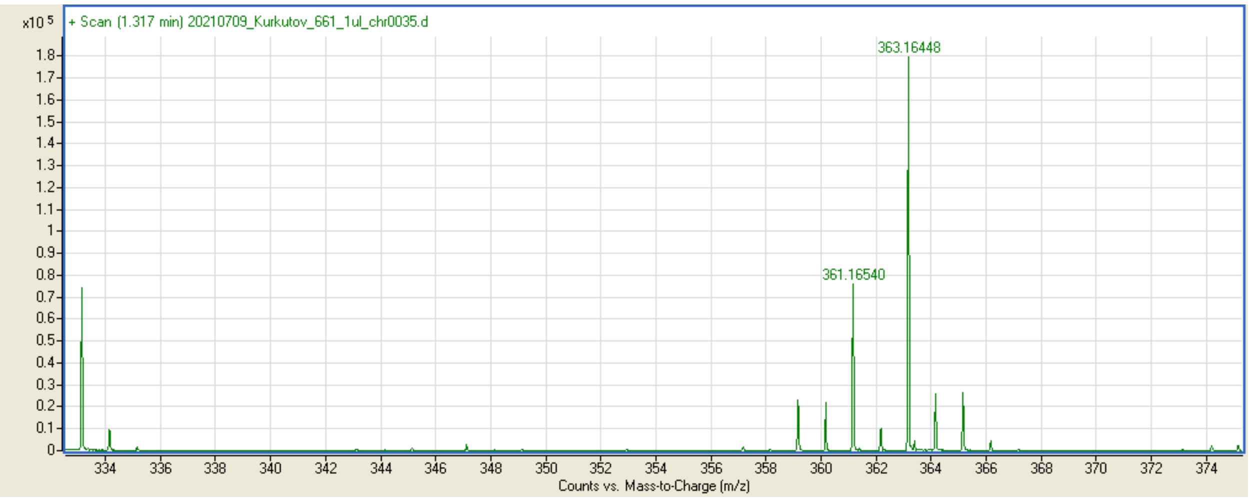

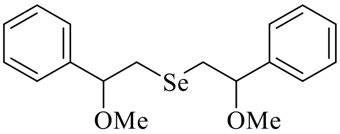

Figure S18. ^{1}H NMR spectrum of compound **3f**

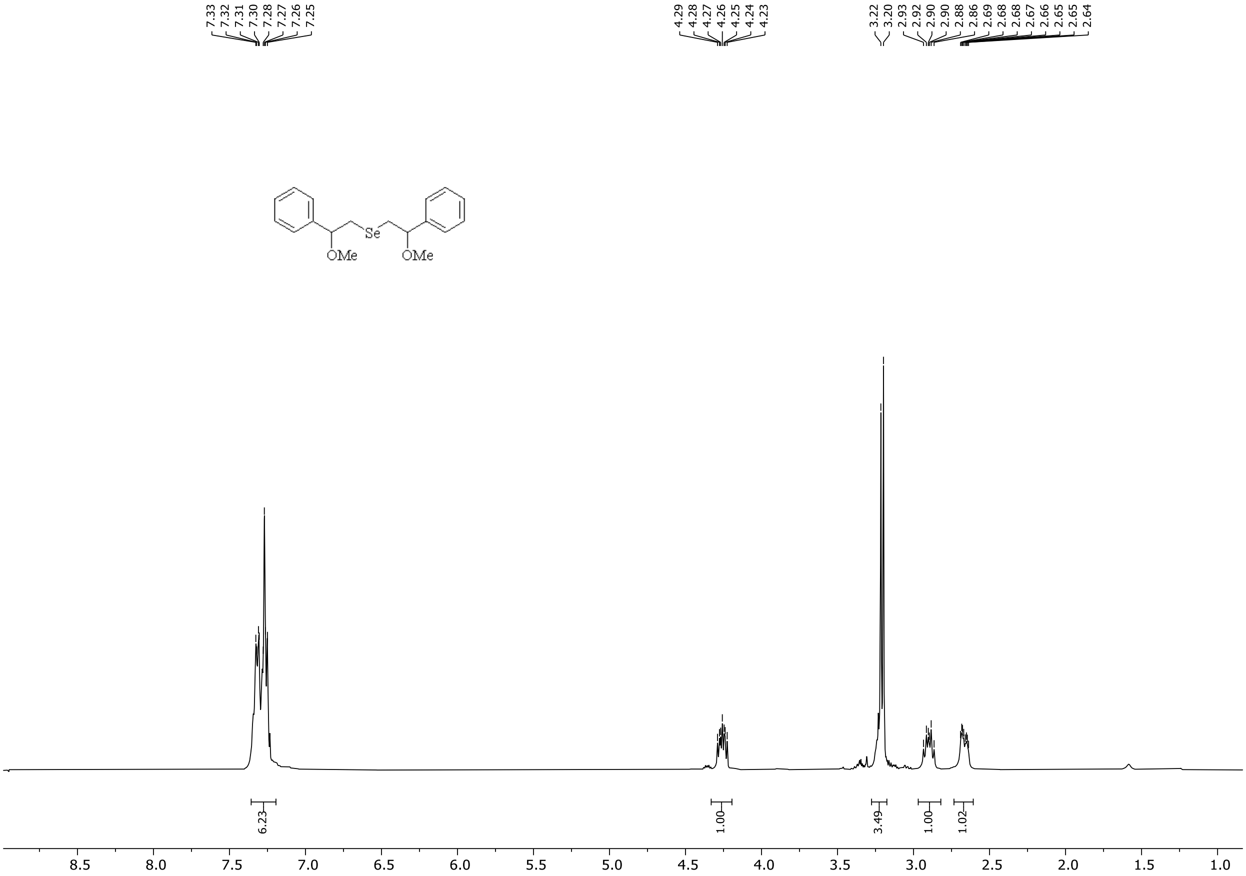

Figure S19. ^{13}C NMR spectrum of compound **3f**

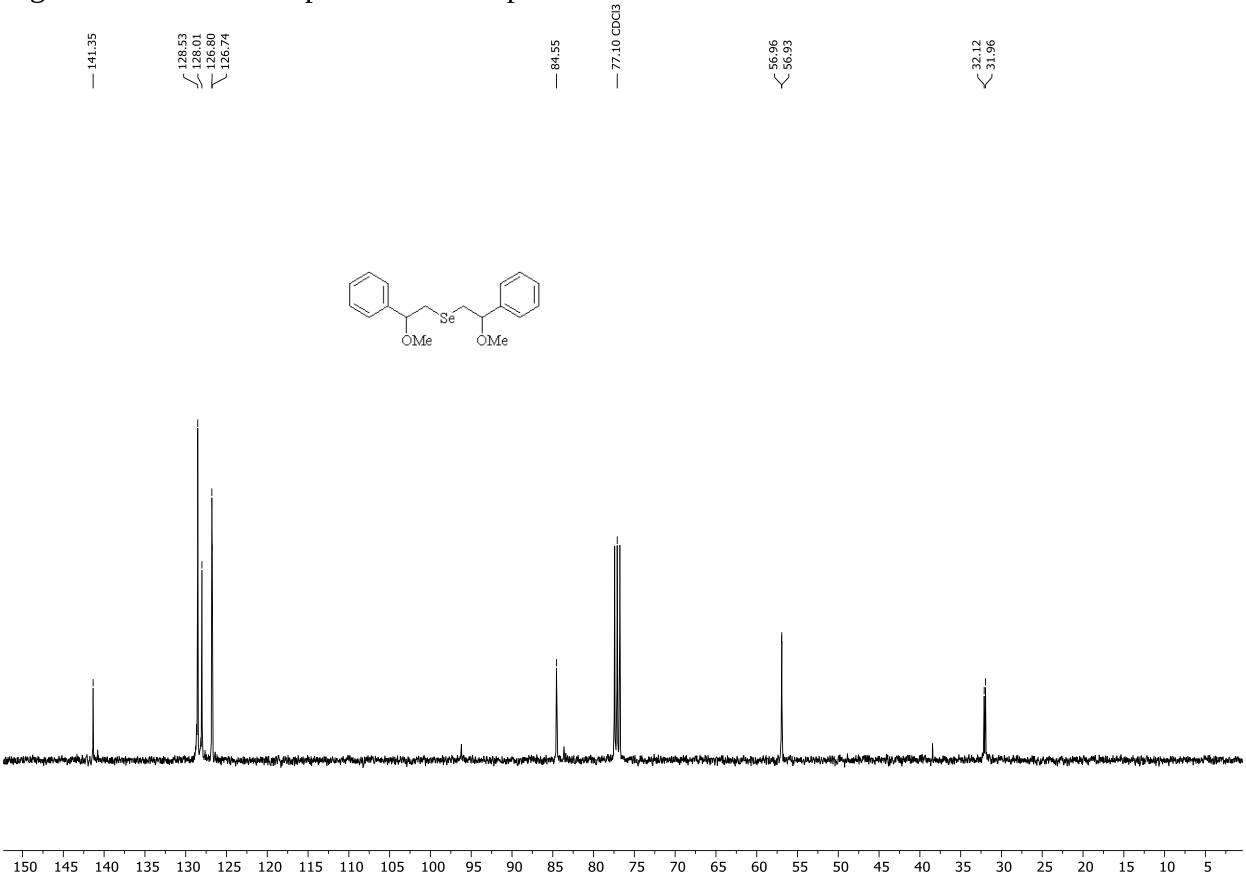

Figure S20. ^{77}Se NMR spectrum of compound **3f**

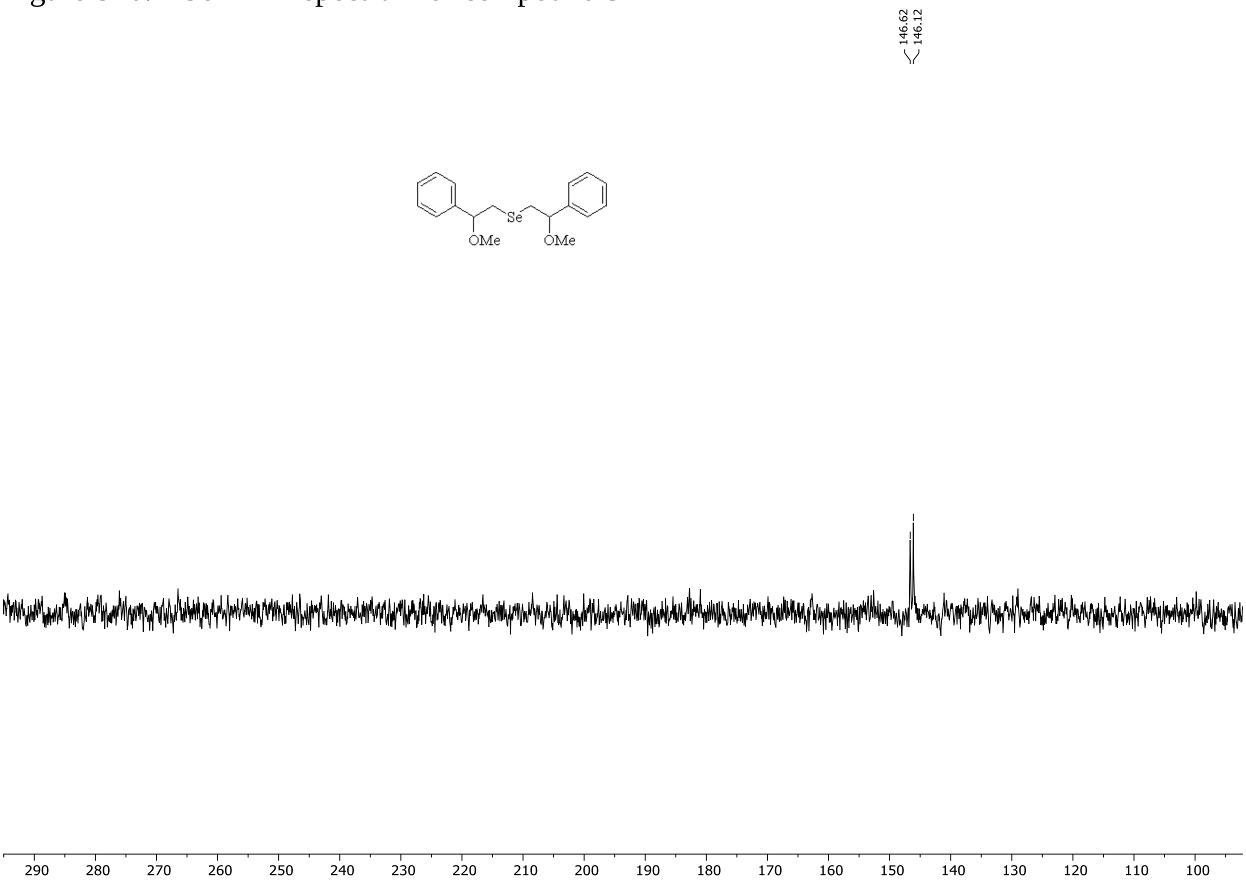

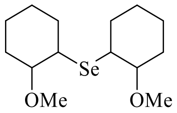

Figure S21. ^{1}H NMR spectrum of compound **3g**

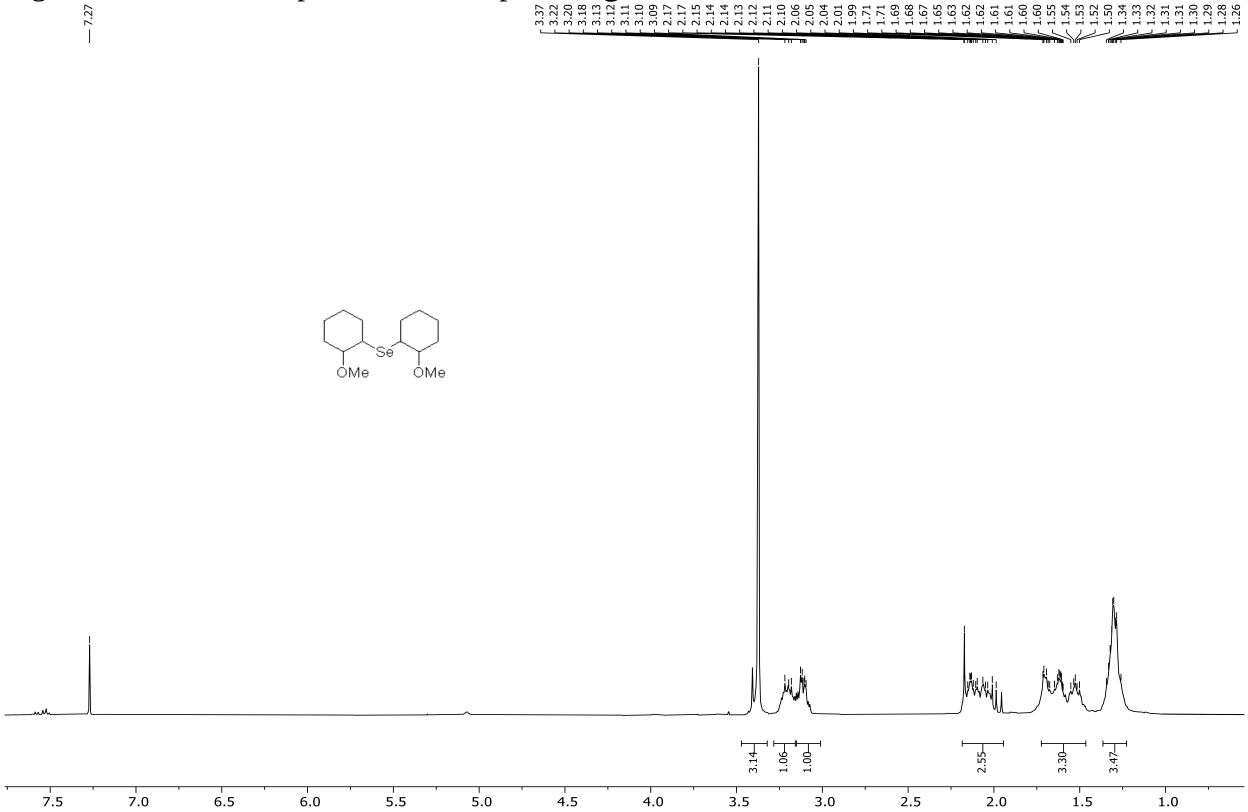

Figure S22. ^{13}C NMR spectrum of compound **3g**

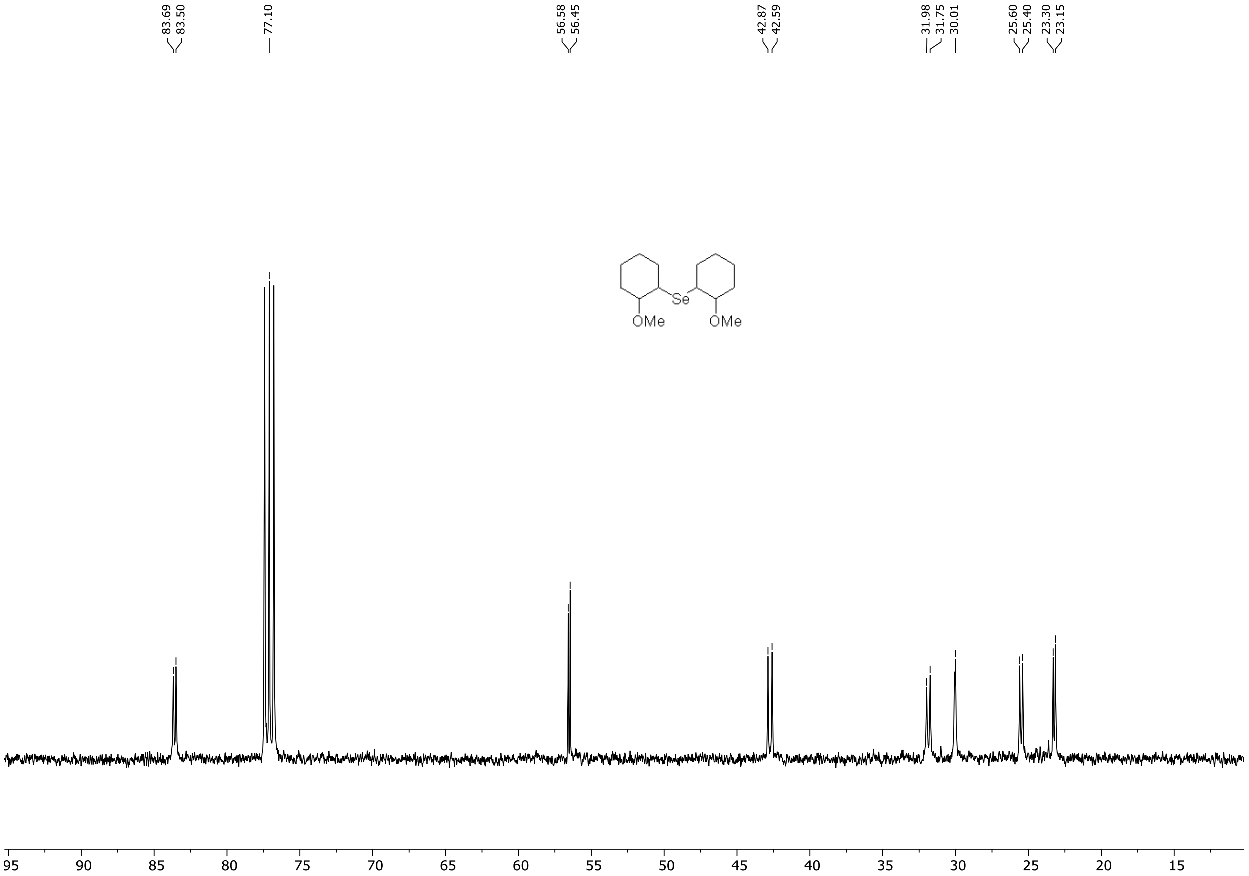

Figure S23. ^{77}Se NMR spectrum of compound **3g**

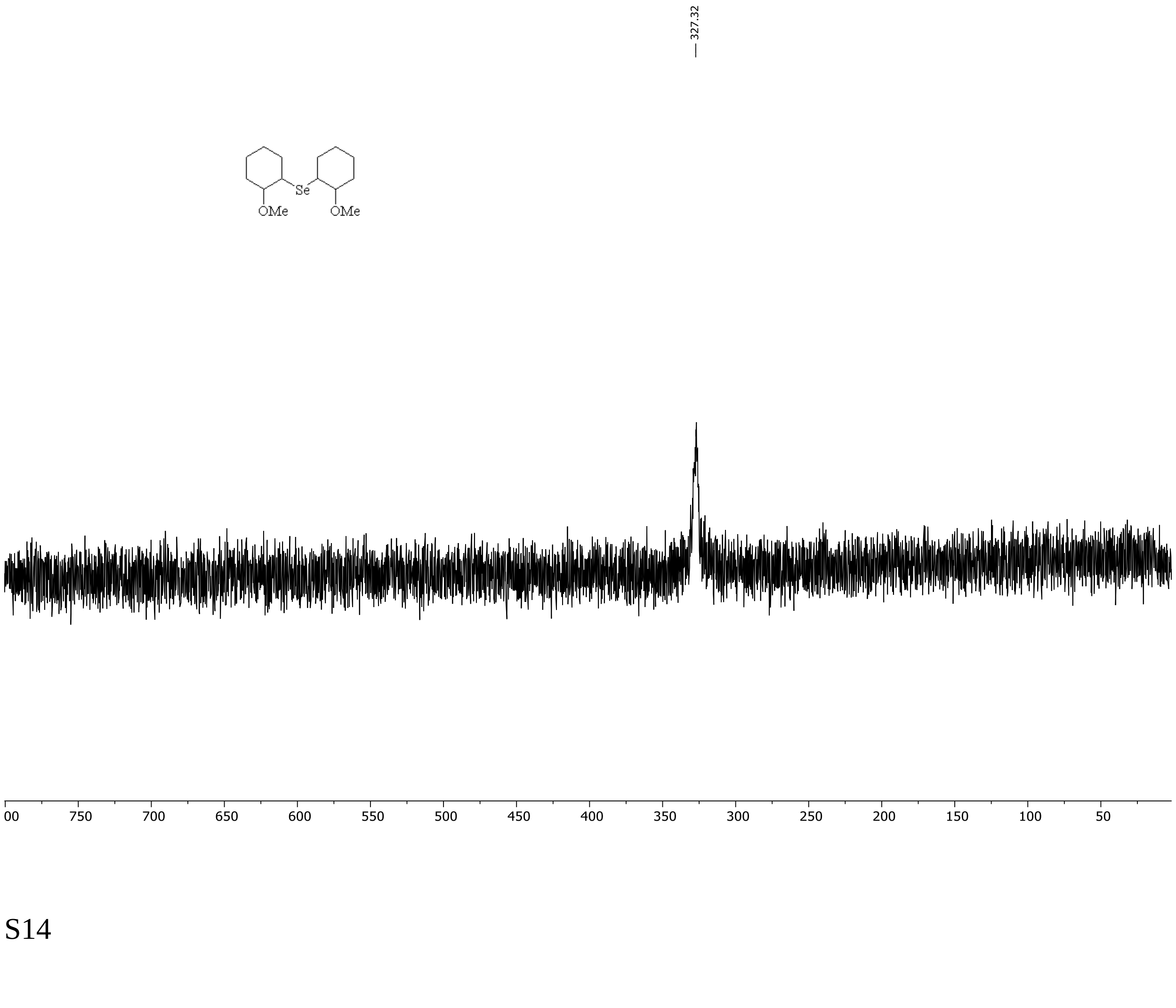

Figure S24. HRMS spectrum of compound **3g**

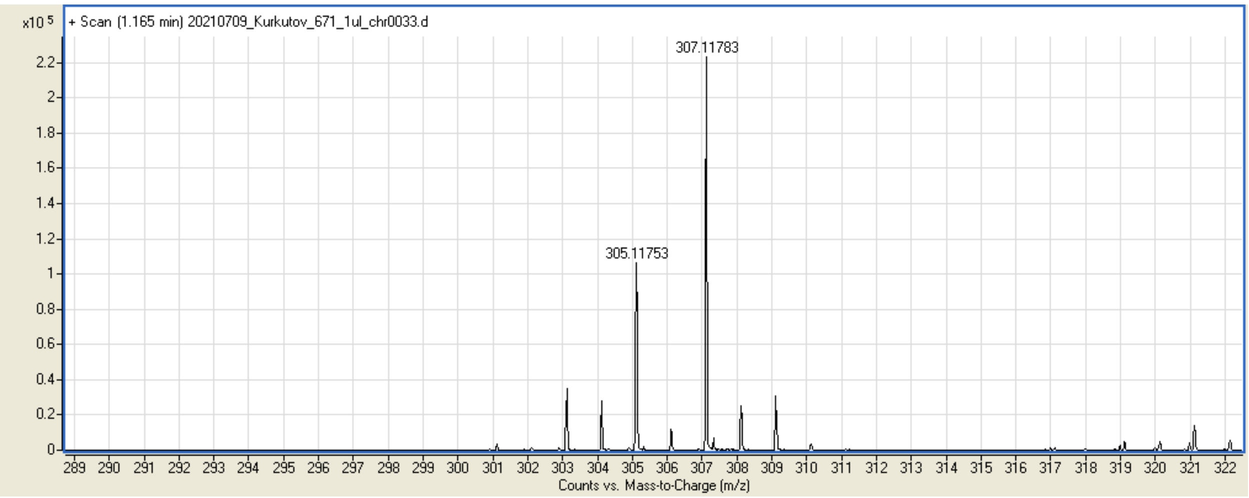

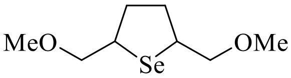

Figure S25. ^{1}H NMR spectrum of compound **5**

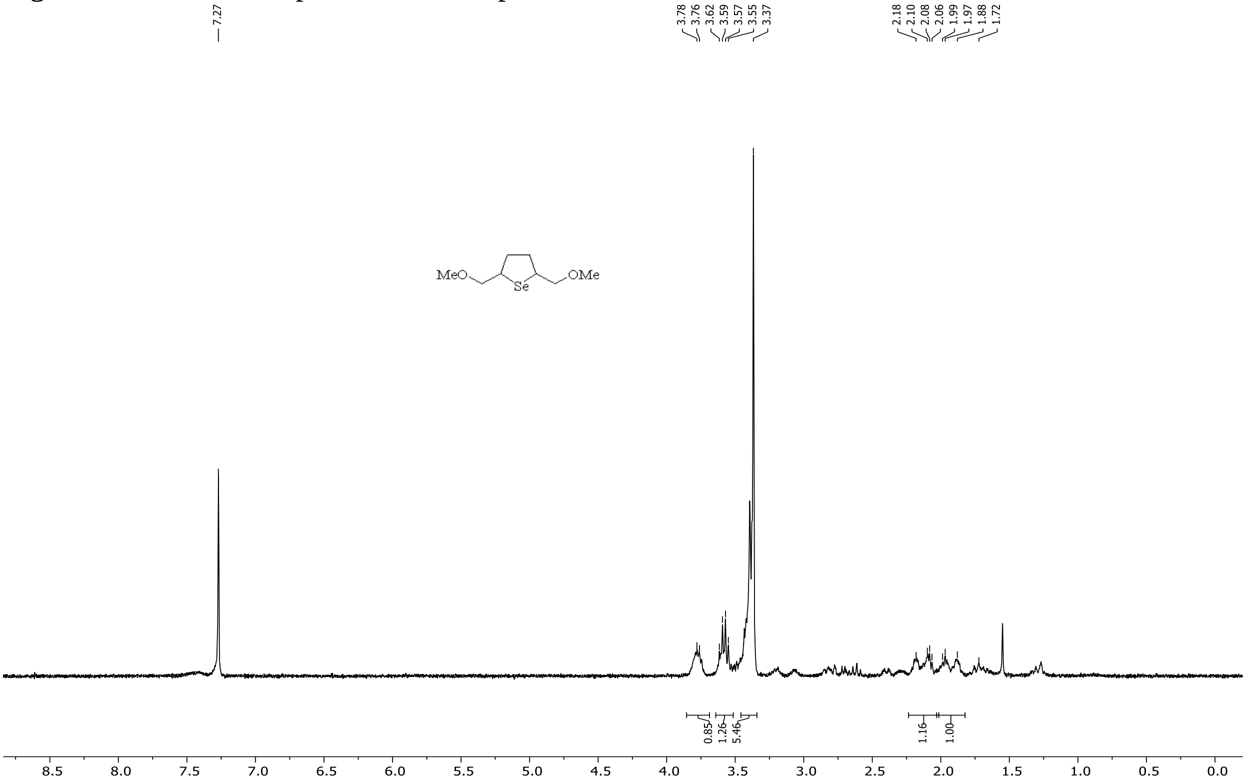

Figure S26. ^{13}C NMR spectrum of compound **5**

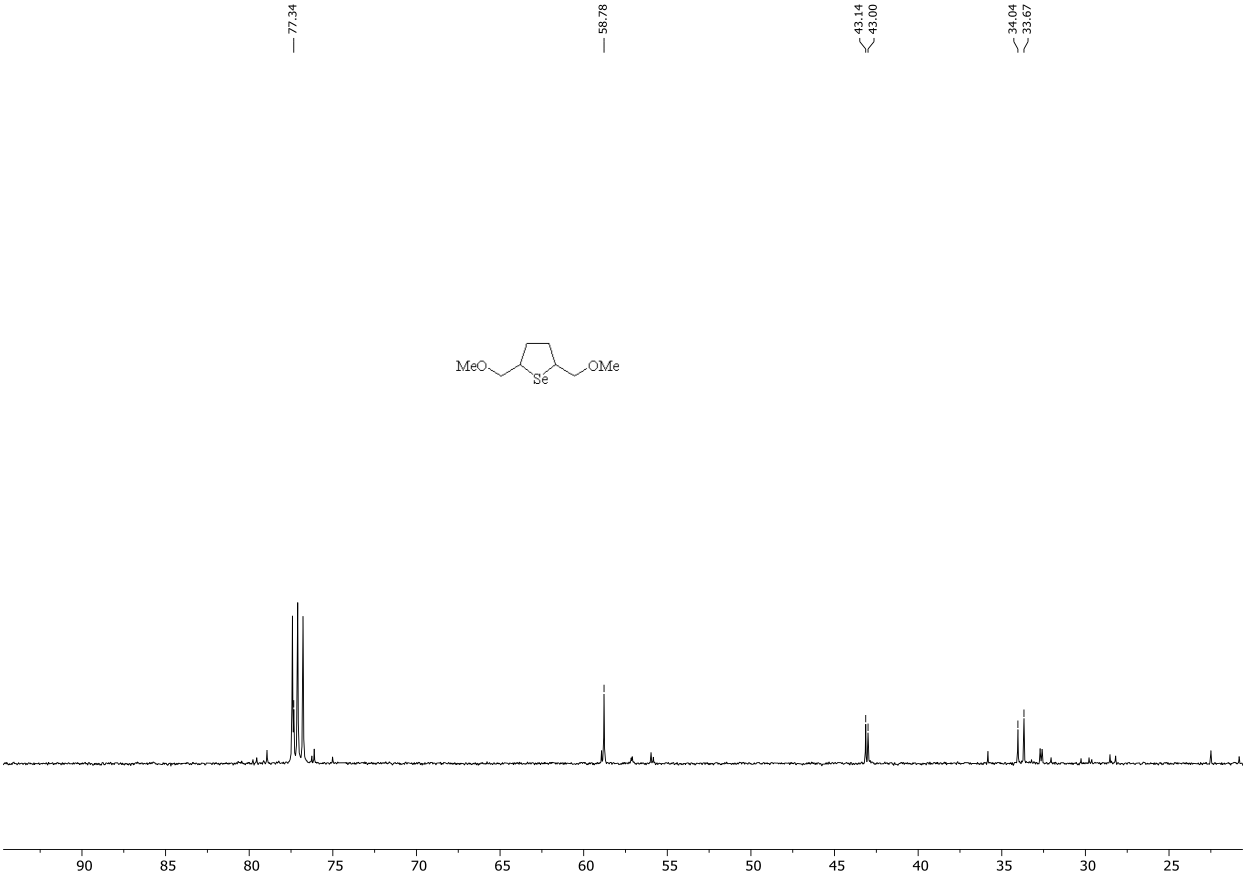

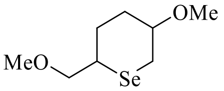

Figure S27. ^{1}H NMR spectrum of compound **6**

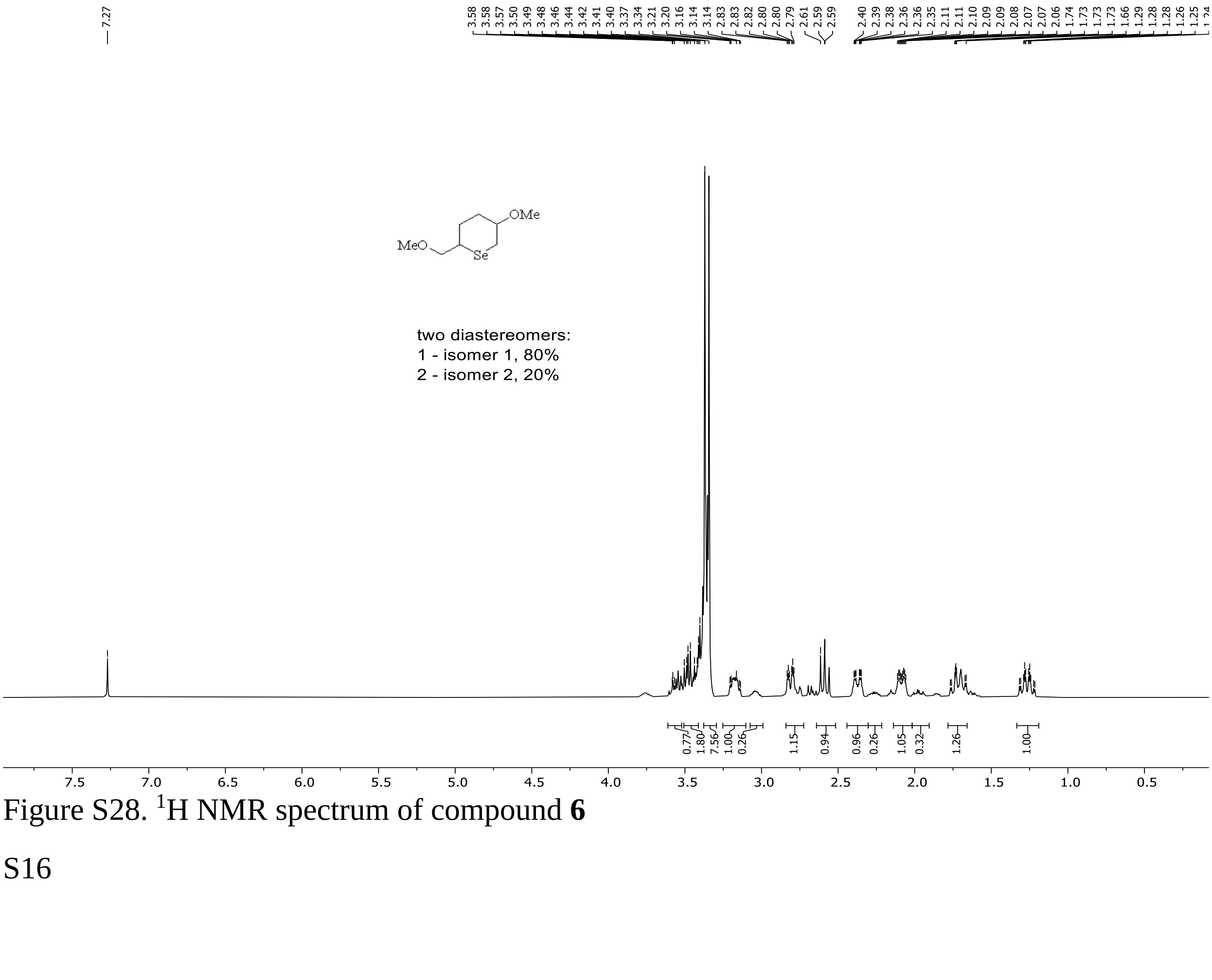

Figure S28. ^{1}H NMR spectrum of compound **6**

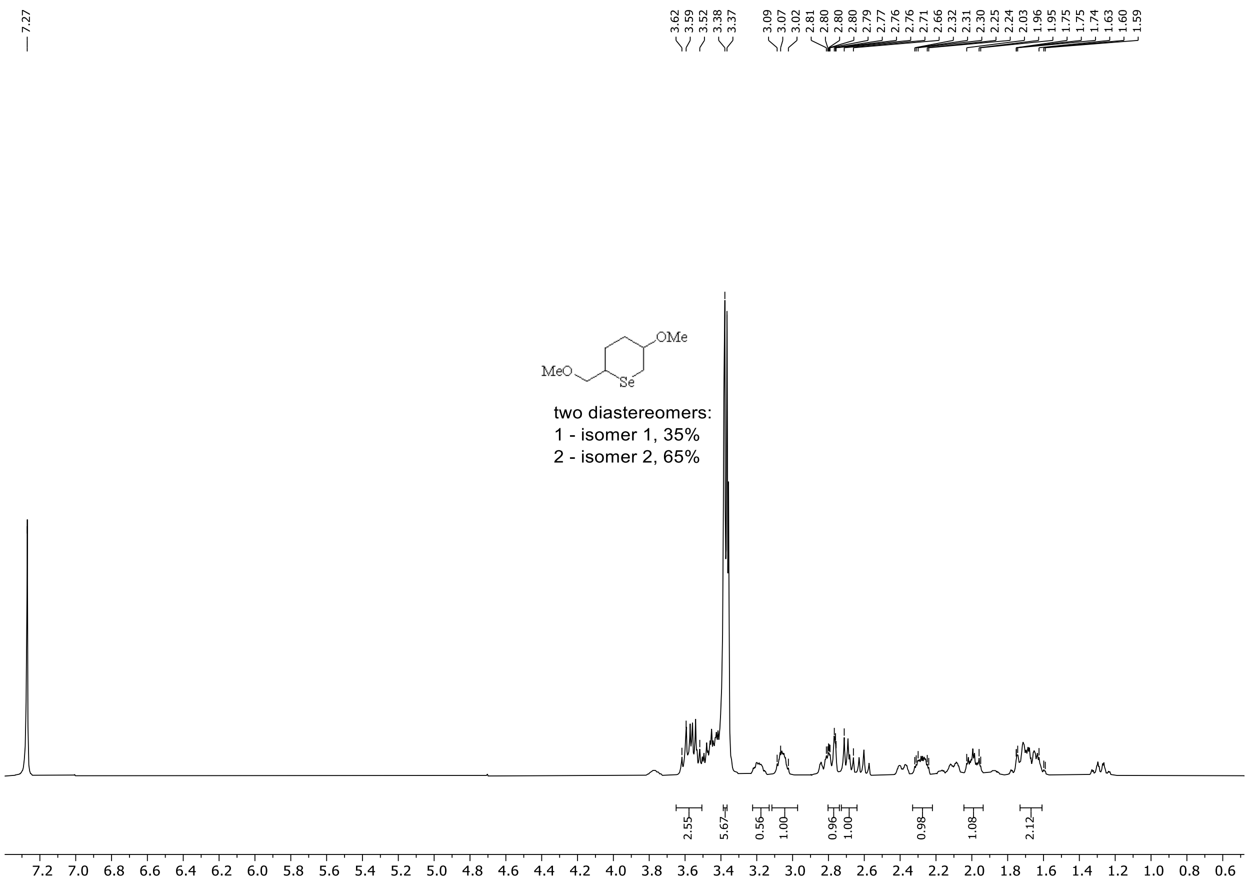

Figure S29. ^{13}C NMR spectrum of compound **6**

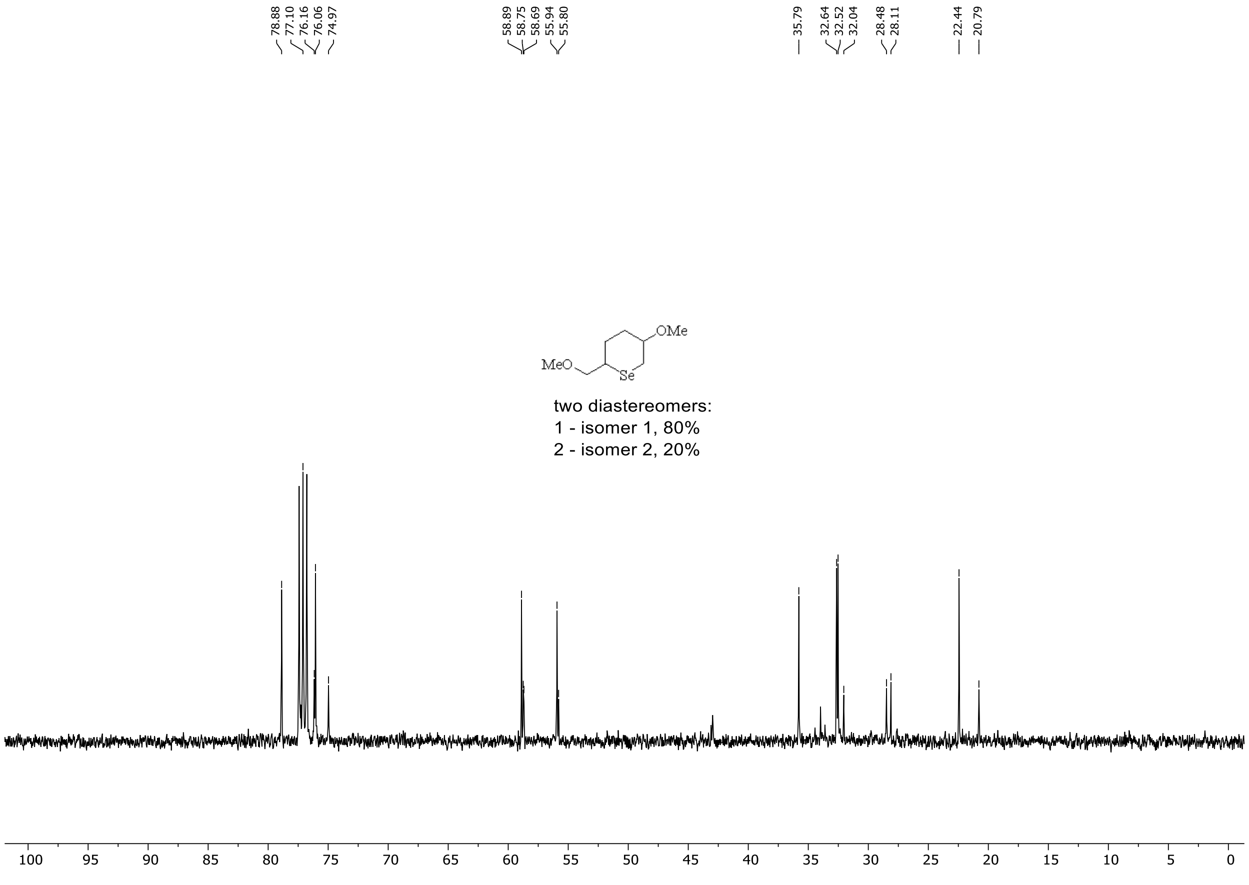

Figure S30. ^{13}C NMR spectrum of compound **6**

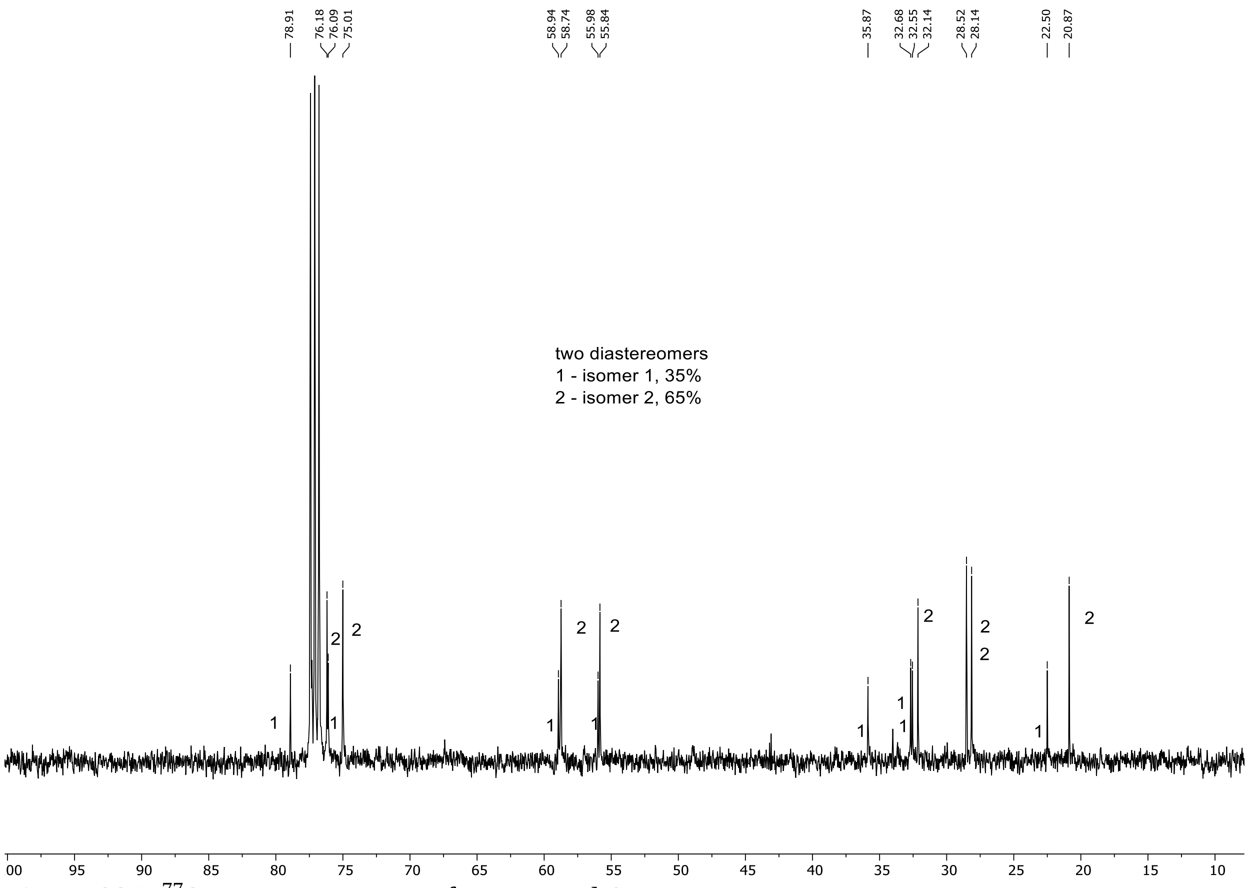

Figure S31. ^{77}Se NMR spectrum of compound **6**

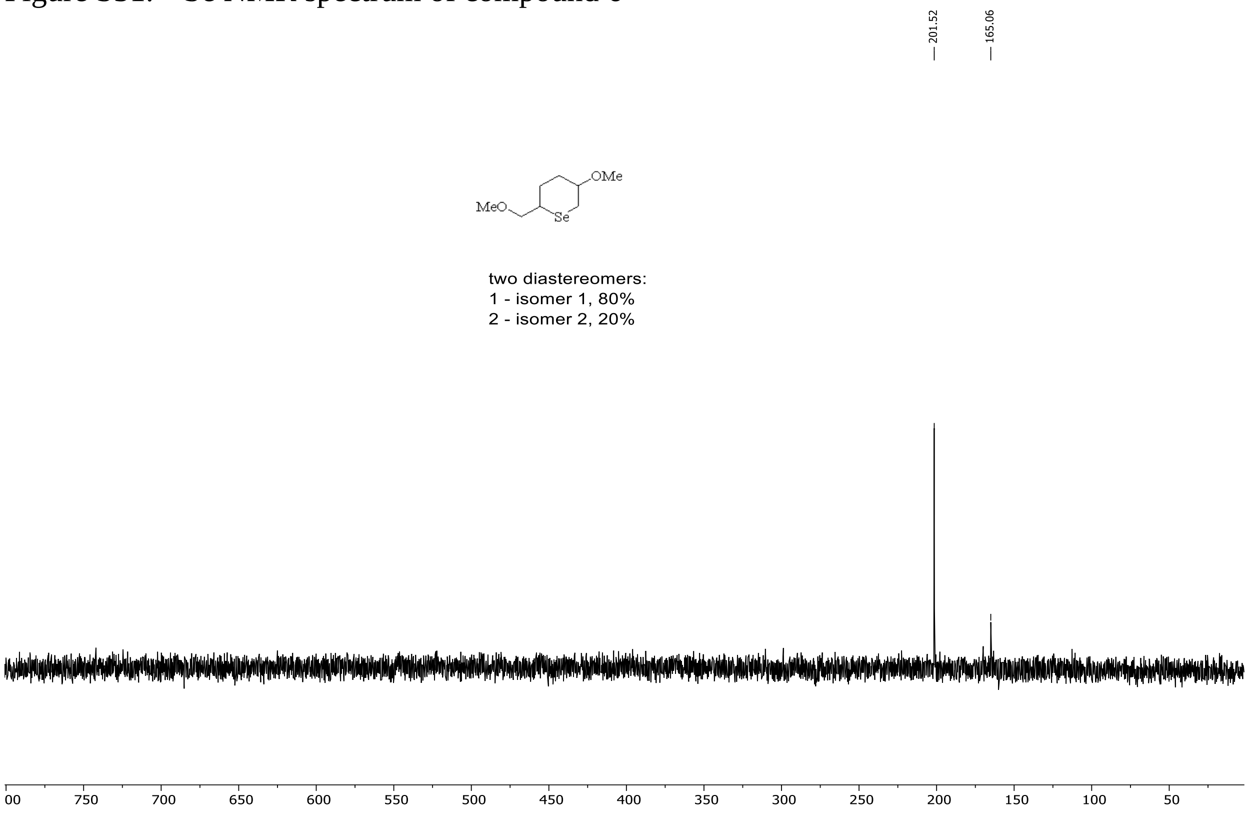

Figure S32. ^{77}Se NMR spectrum of mixture of compounds **5** and **6**

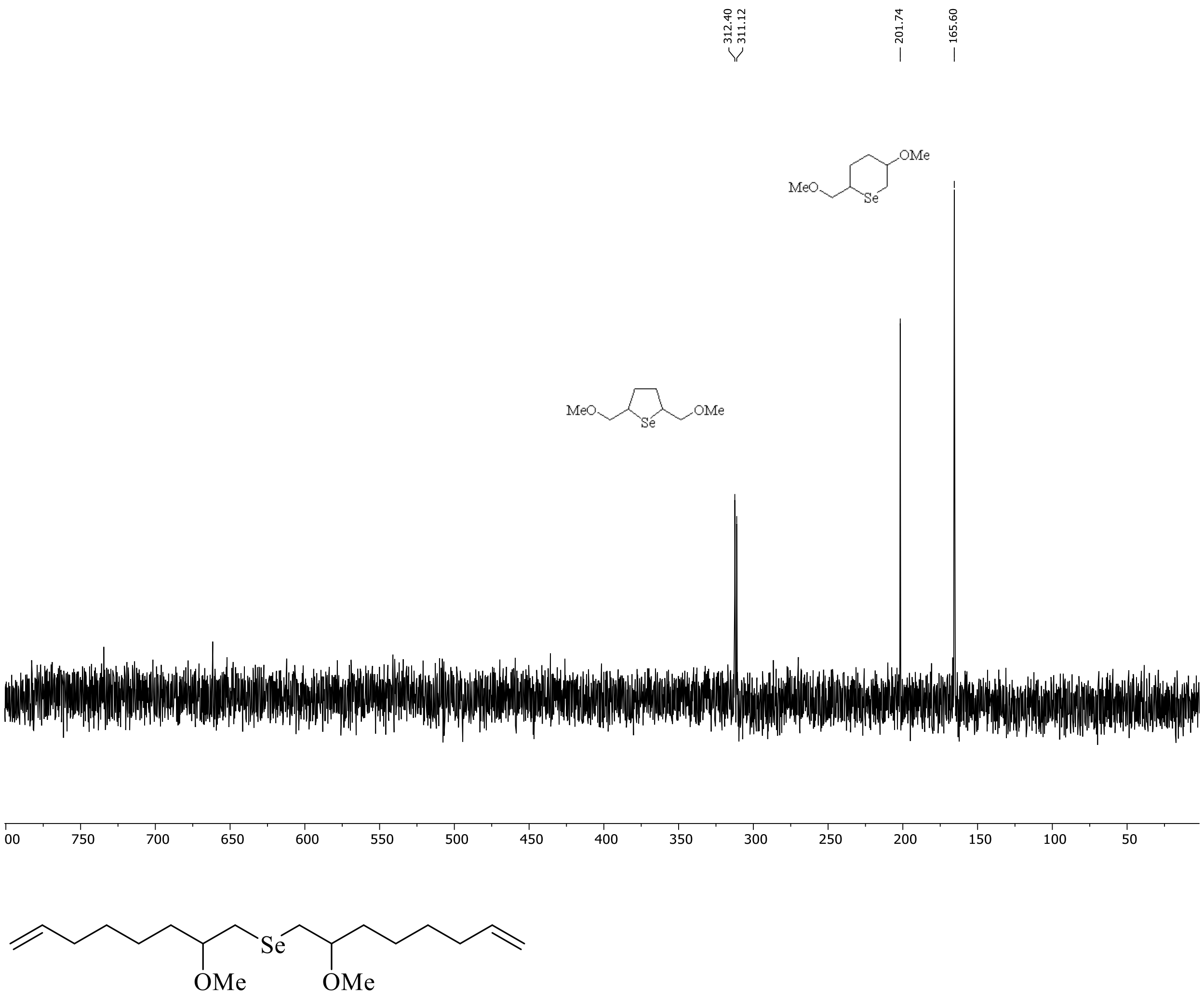

Figure S33. ^{1}H NMR spectrum of compound **7**

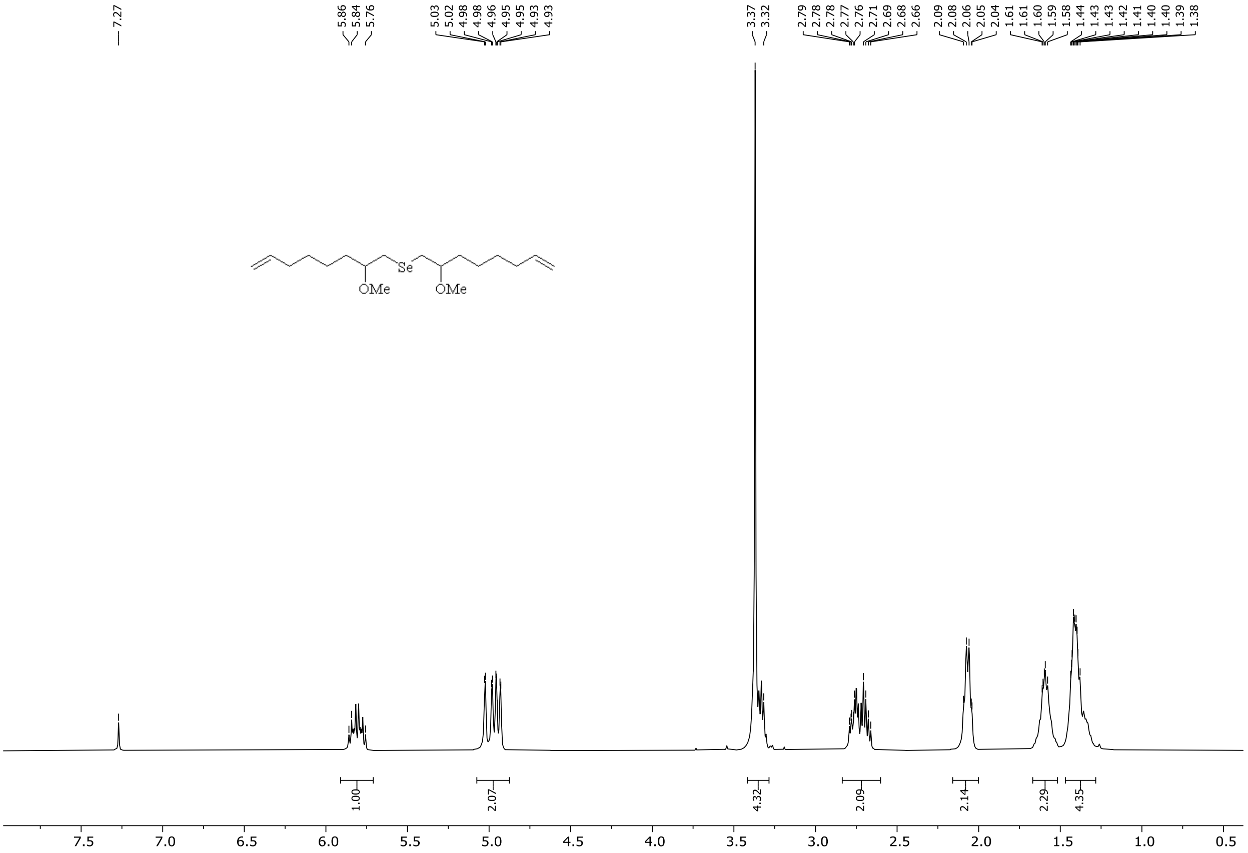

Figure S34. ^{13}C NMR spectrum of compound **7**

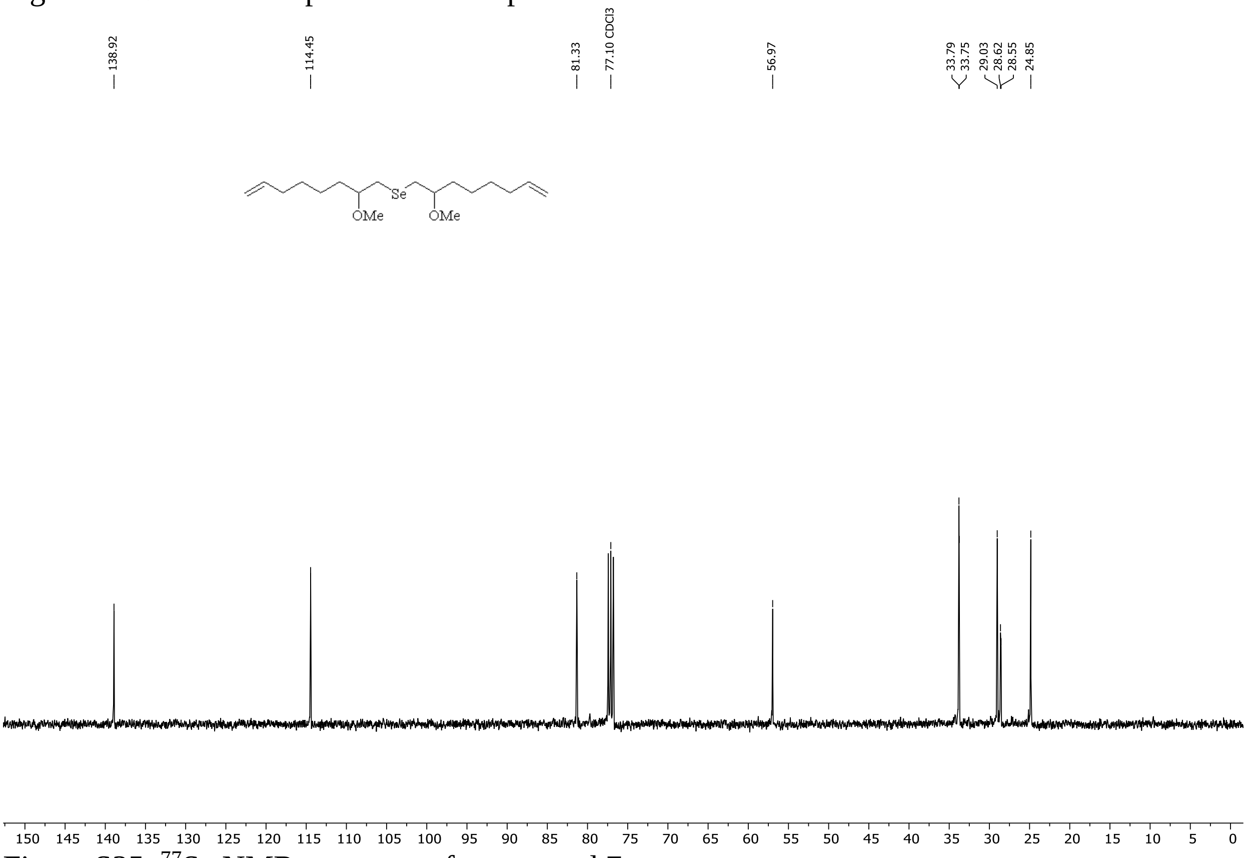

Figure S35. ^{77}Se NMR spectrum of compound **7**

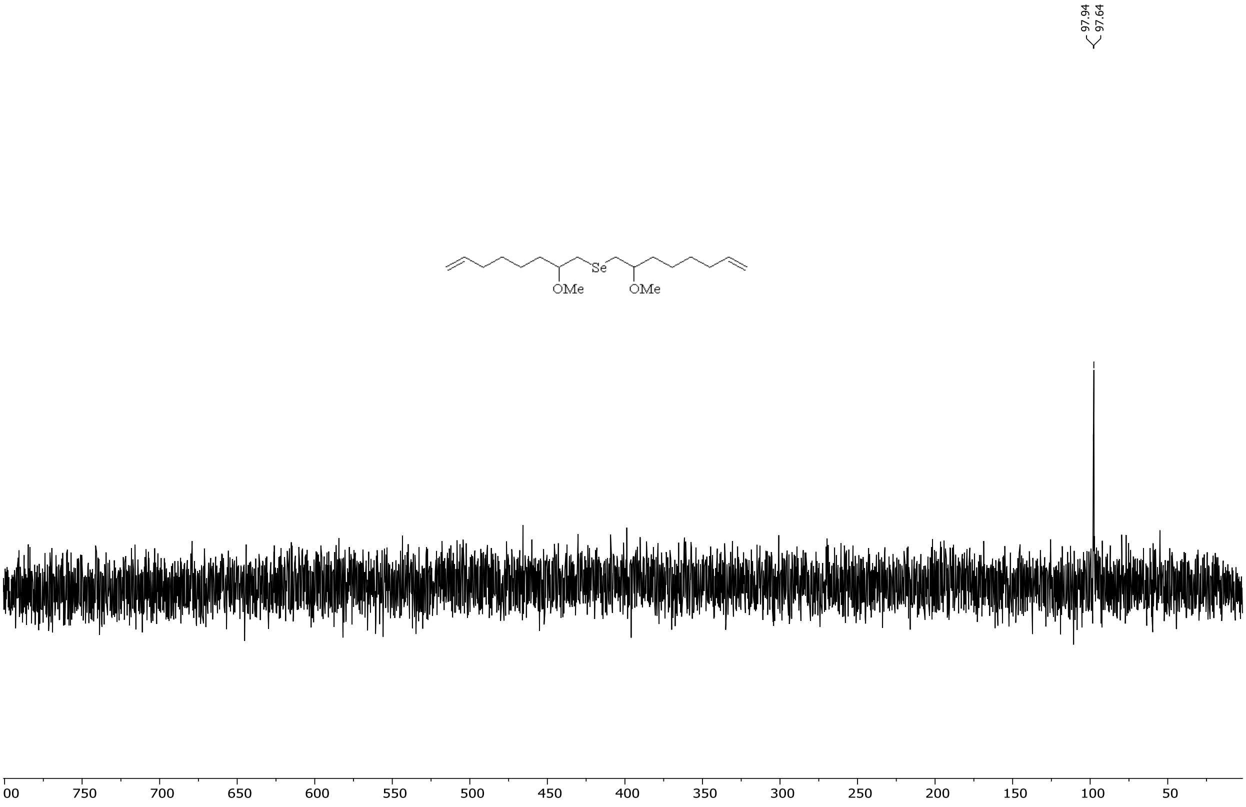

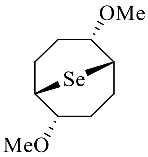

Figure S36. ^{1}H NMR spectrum of compound **9a**

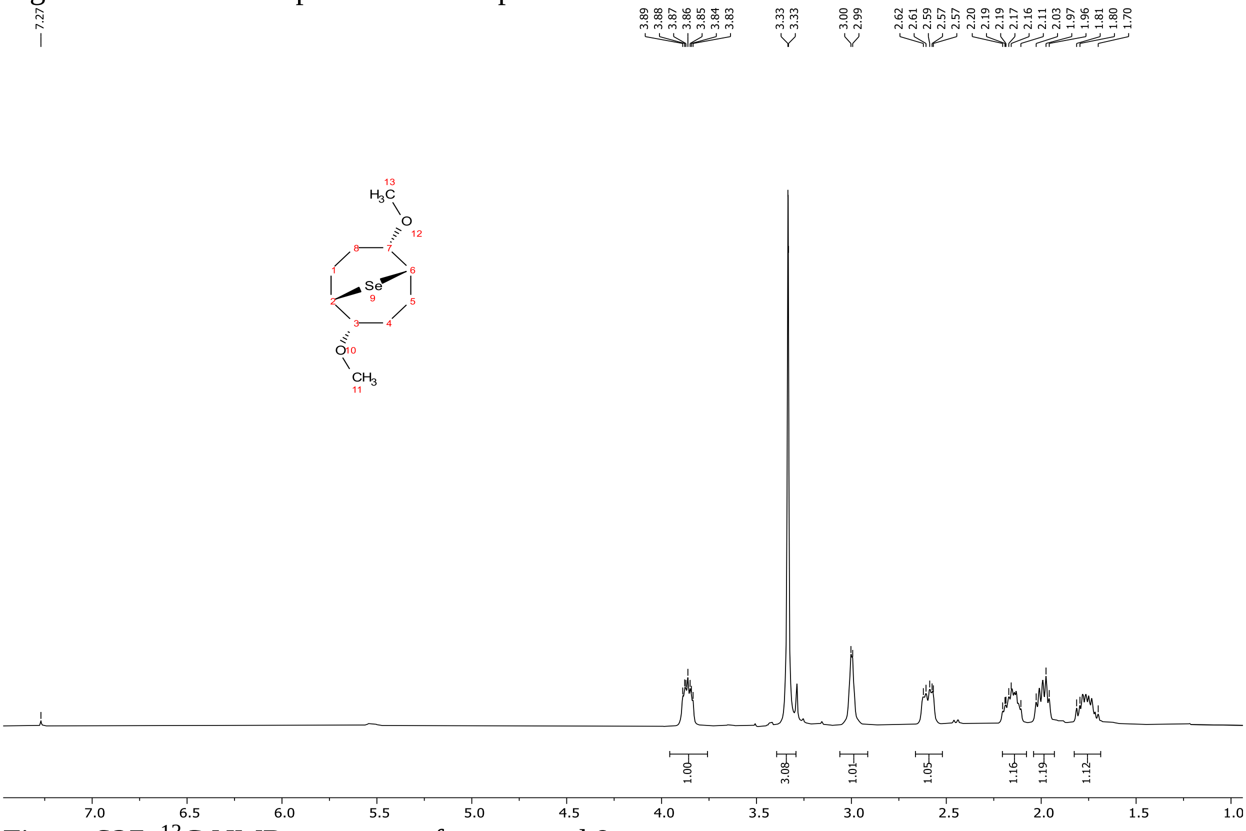

Figure S37. ^{13}C NMR spectrum of compound **9a**

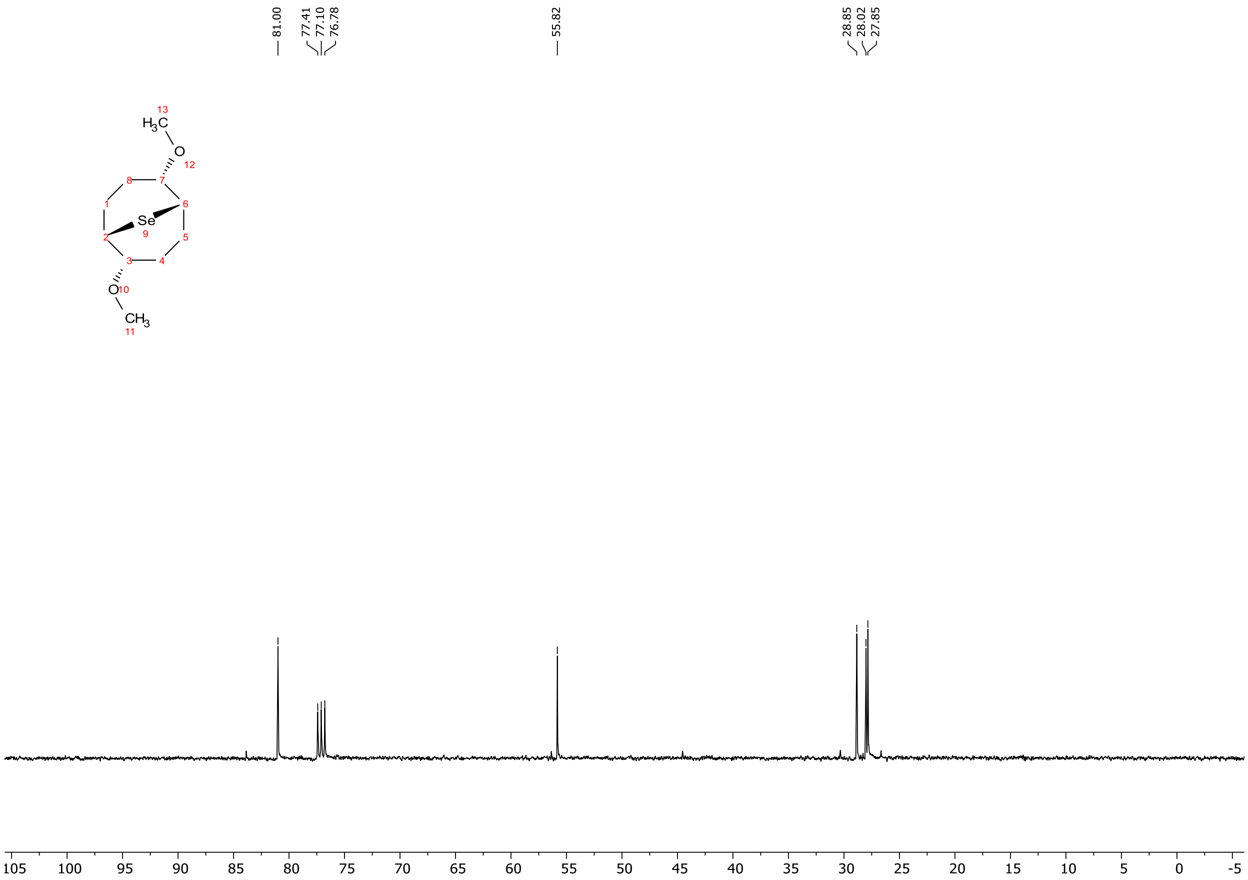

Figure S38. ^{77}Se NMR spectrum of compound **9a**

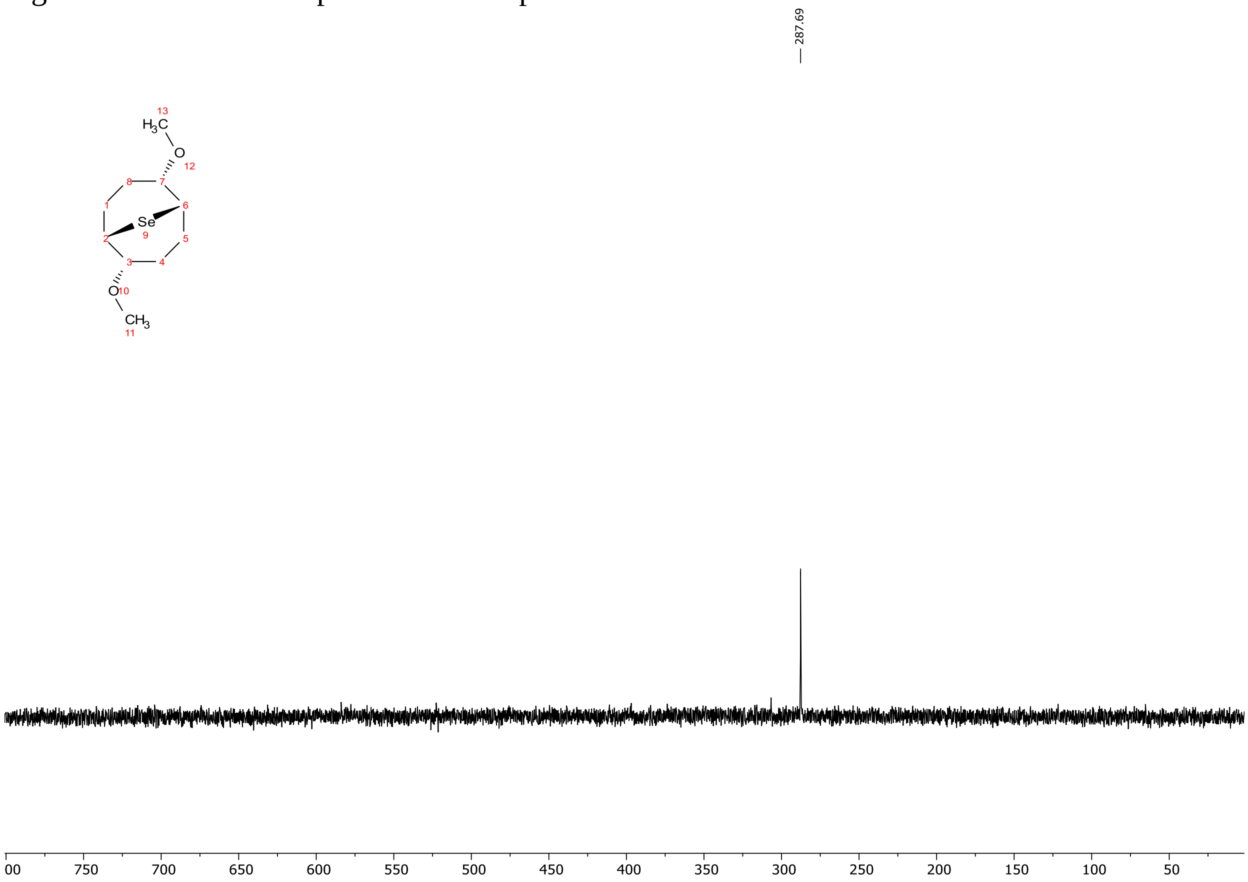

Figure S39. ^{1}H NMR spectrum of compound **9b**

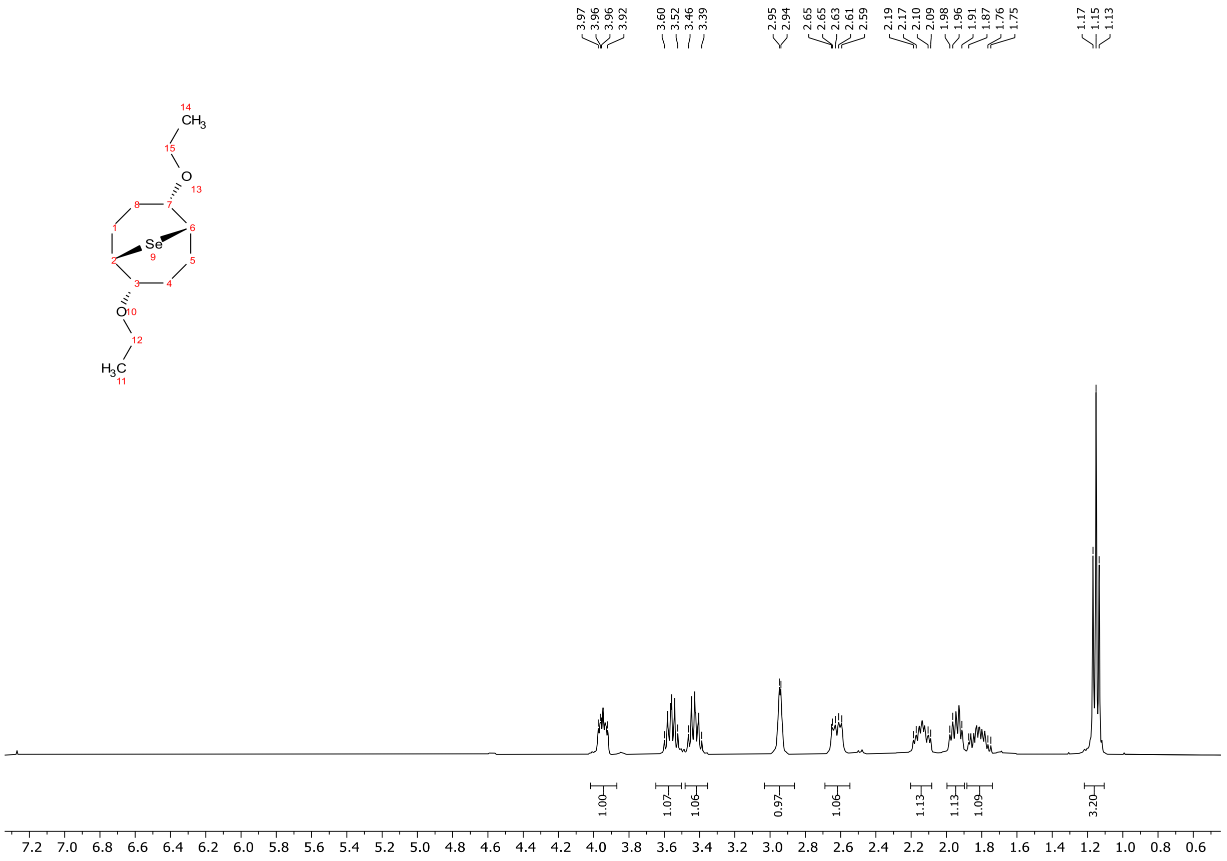

Figure S40. ^{13}C NMR spectrum of compound **9b**

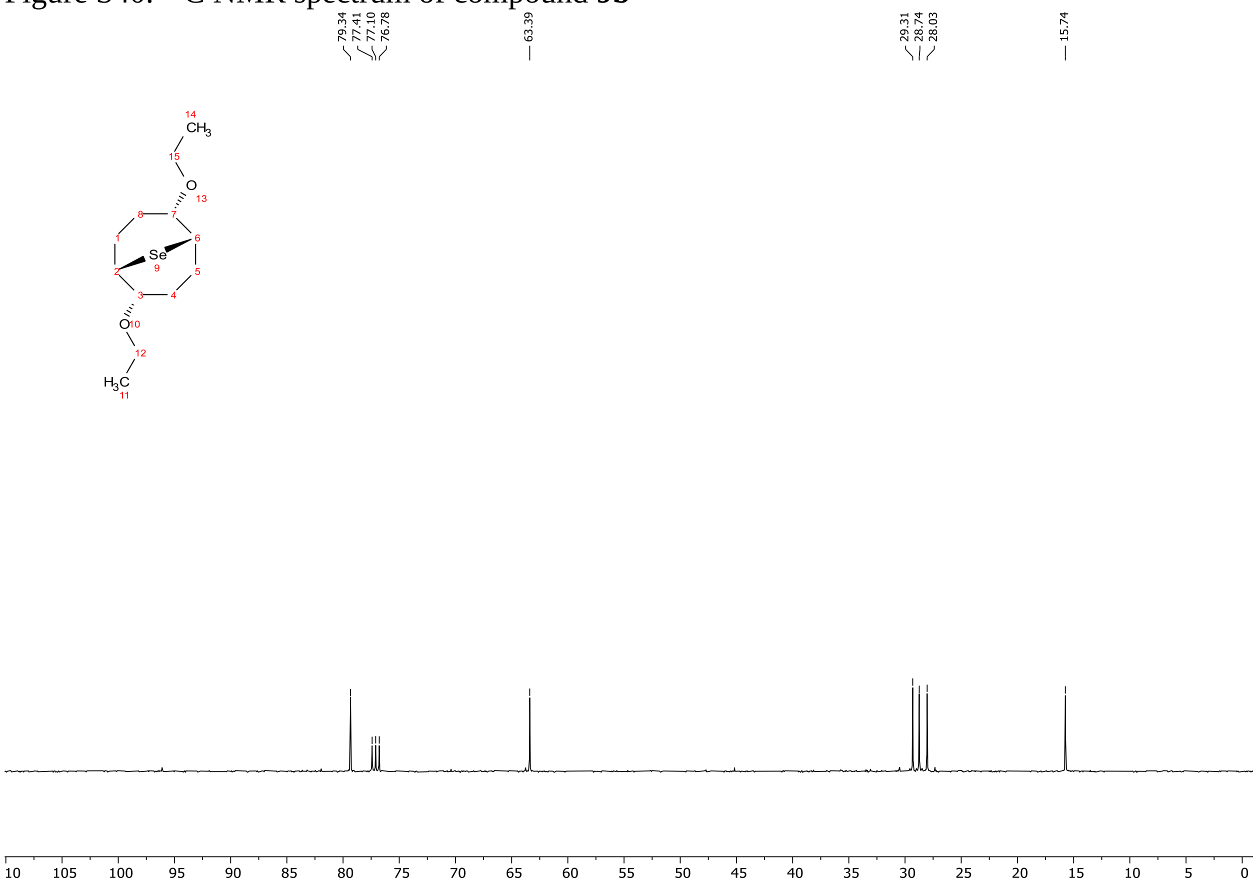

Figure S41. ^{77}SeNMR spectrum of compound **9b**

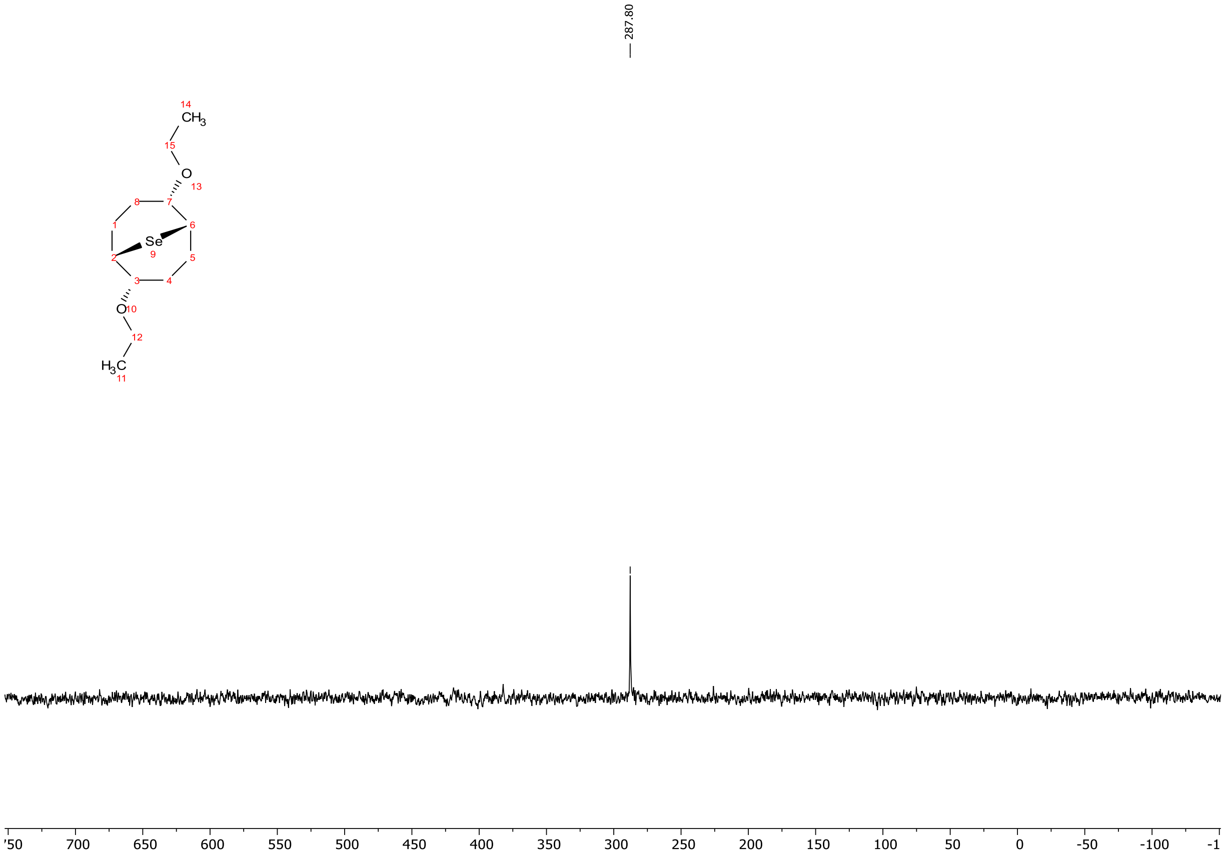

Figure S42. ^{1}H NMR spectrum of compound **9c**

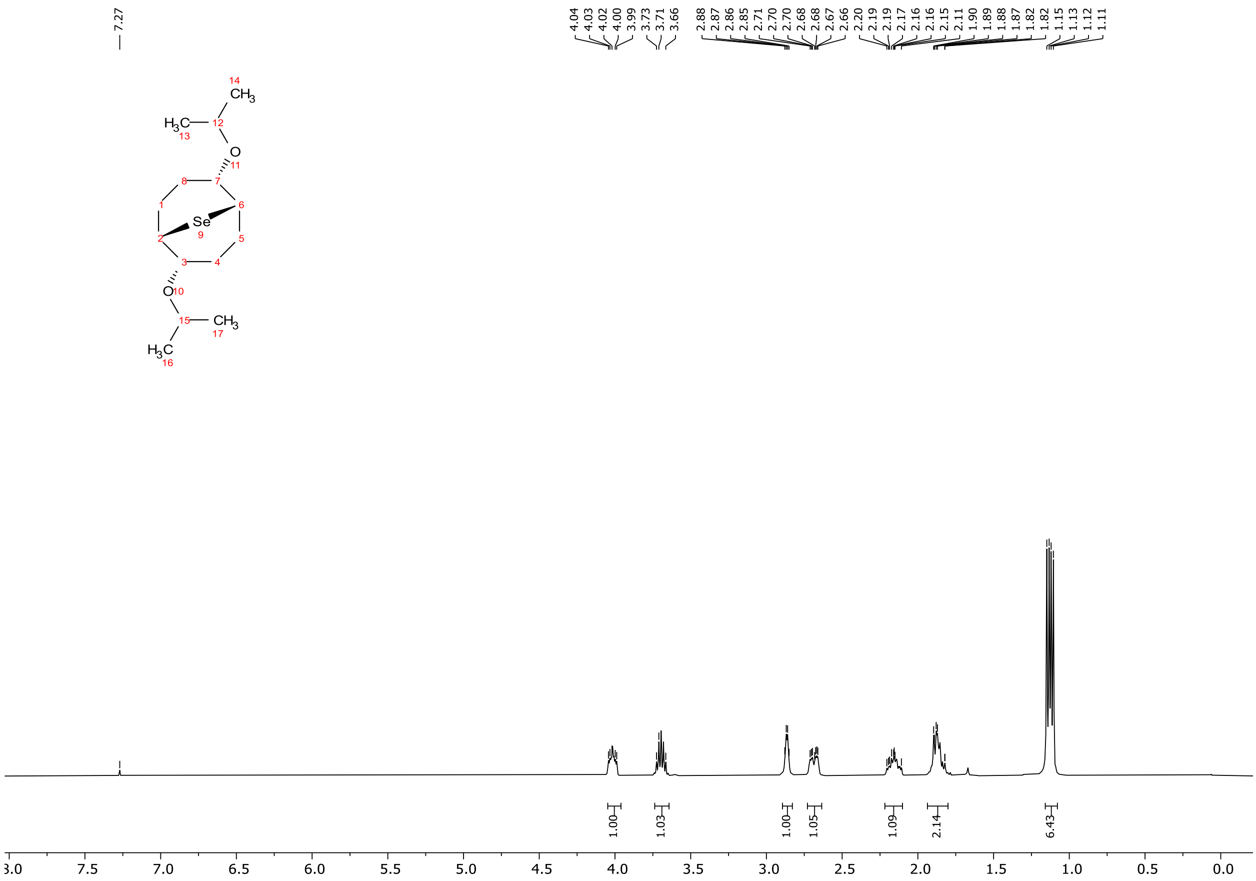

Figure S43. ^{13}C NMR spectrum of compound **9c**

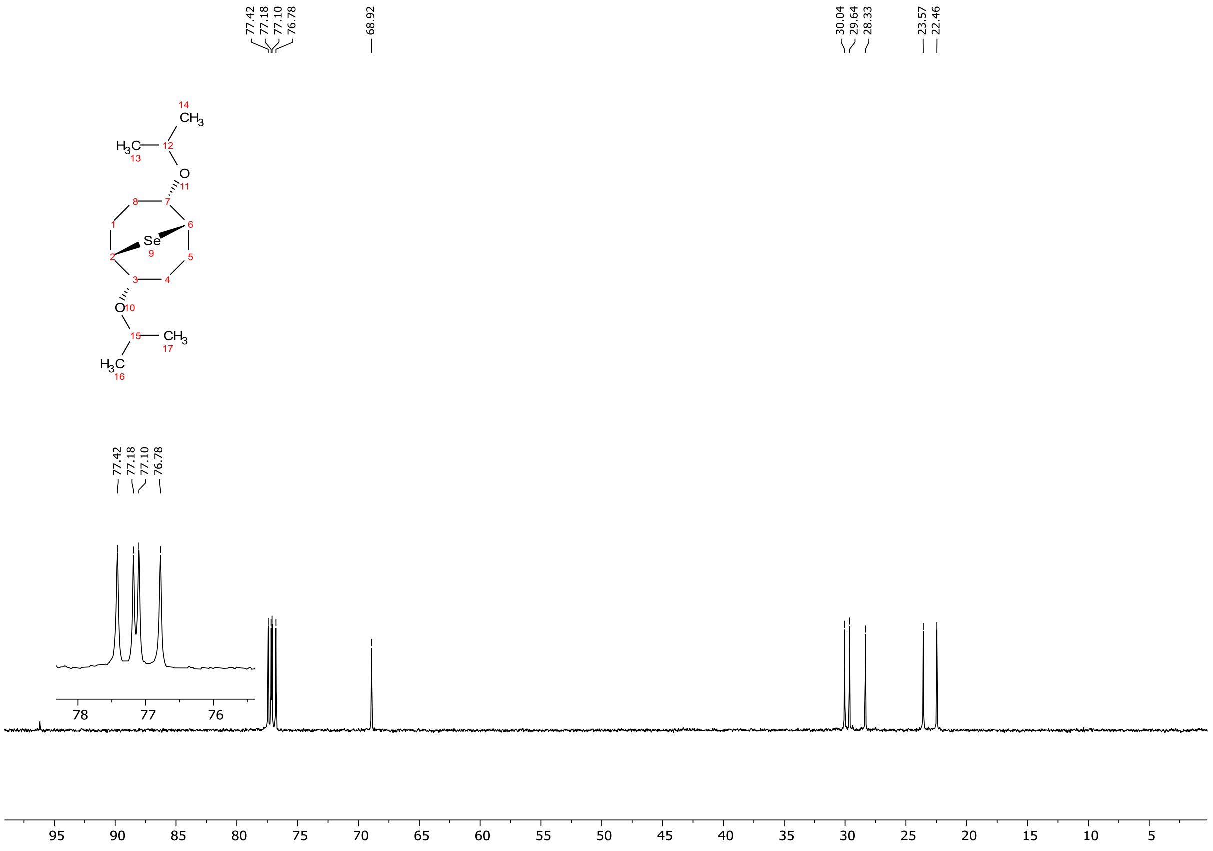

Figure S44. ^{77}Se NMR spectrum of compound **9c**

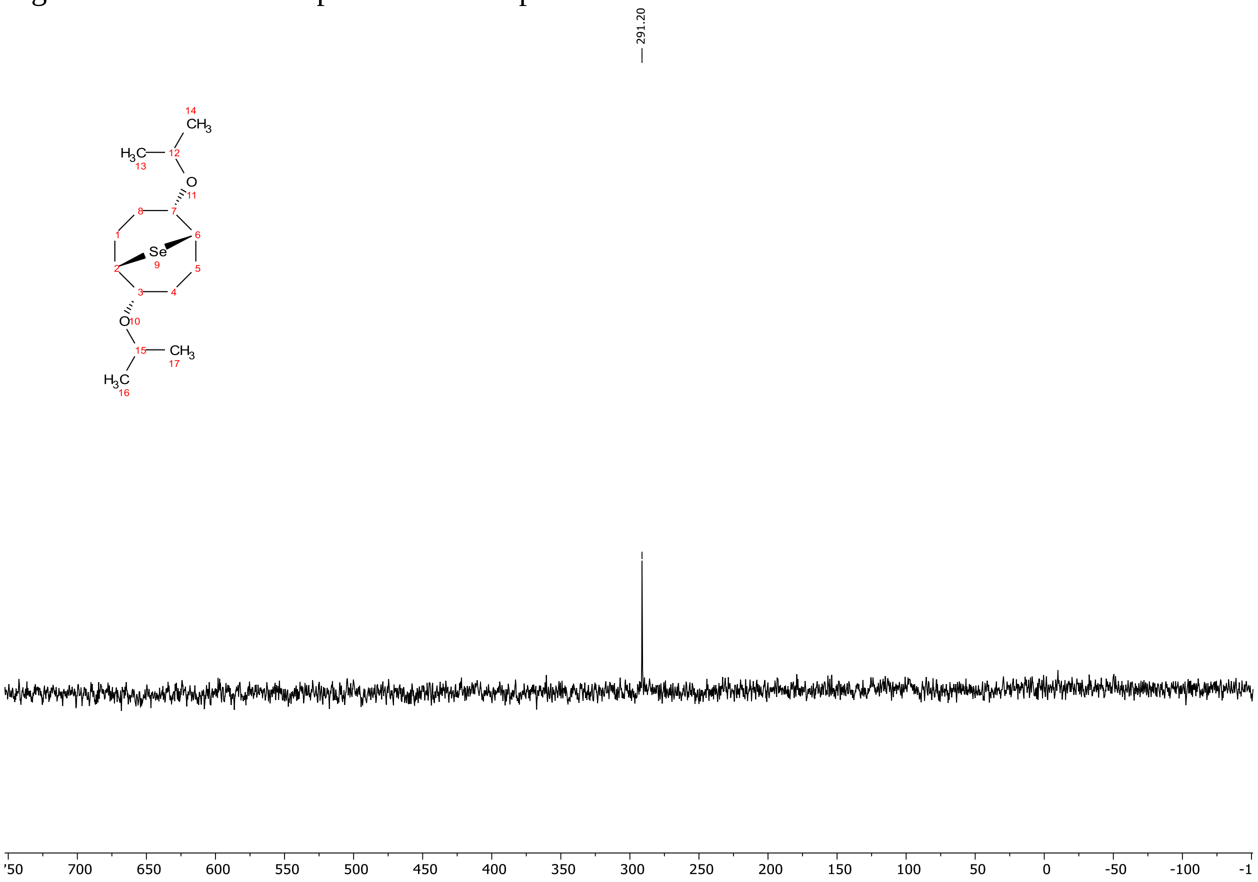

Figure S45. ^{1}H NMR spectrum of compound **10**

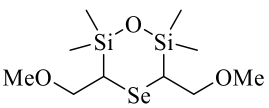

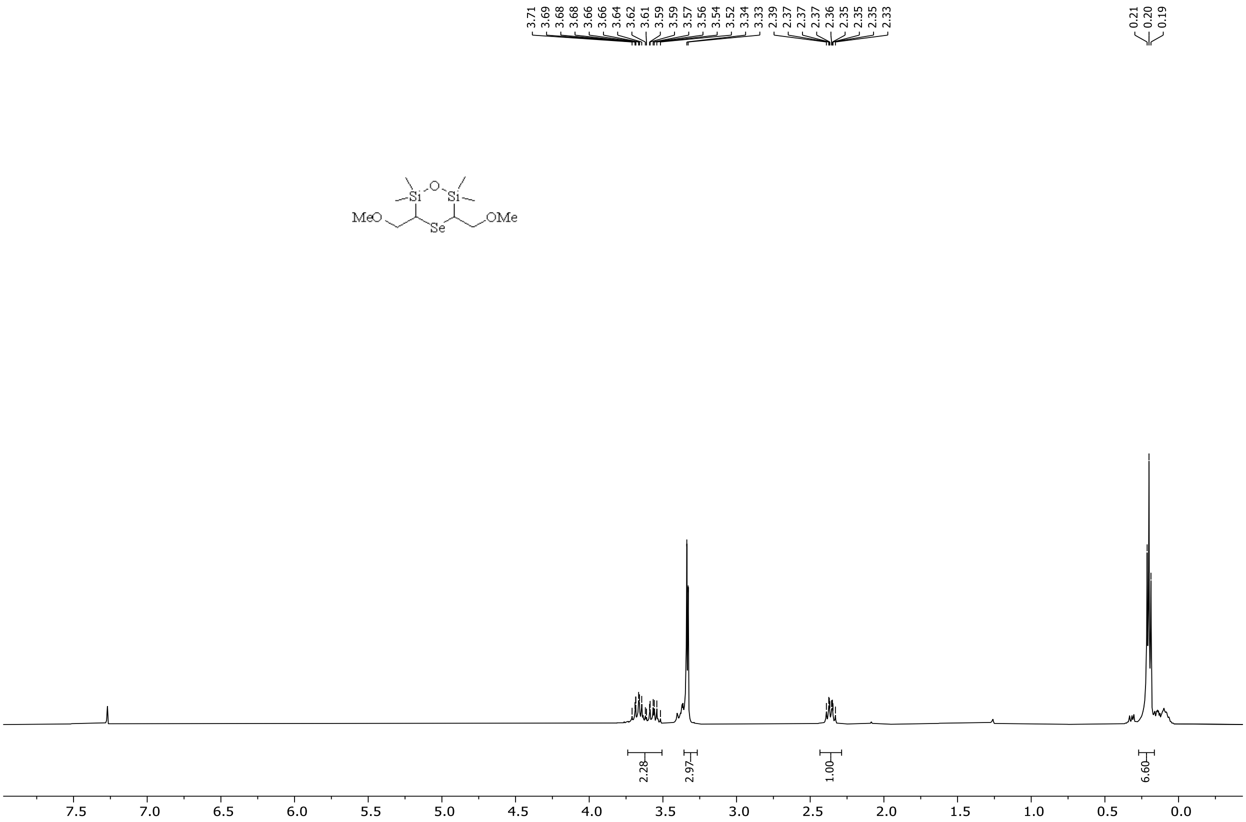

Figure S46. ^{13}C NMR spectrum of compound **10**

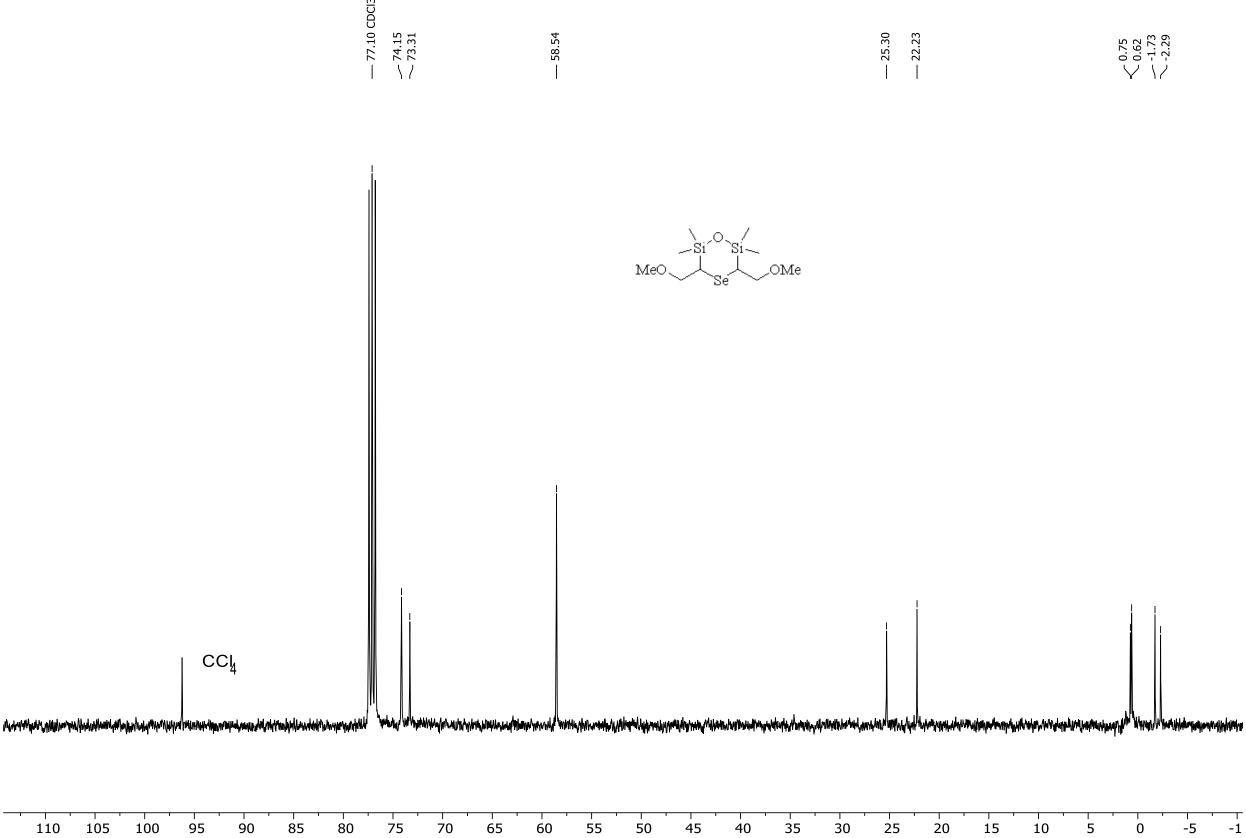

Figure S47. ^{13}C NMR j-mod spectrum of compound **10**

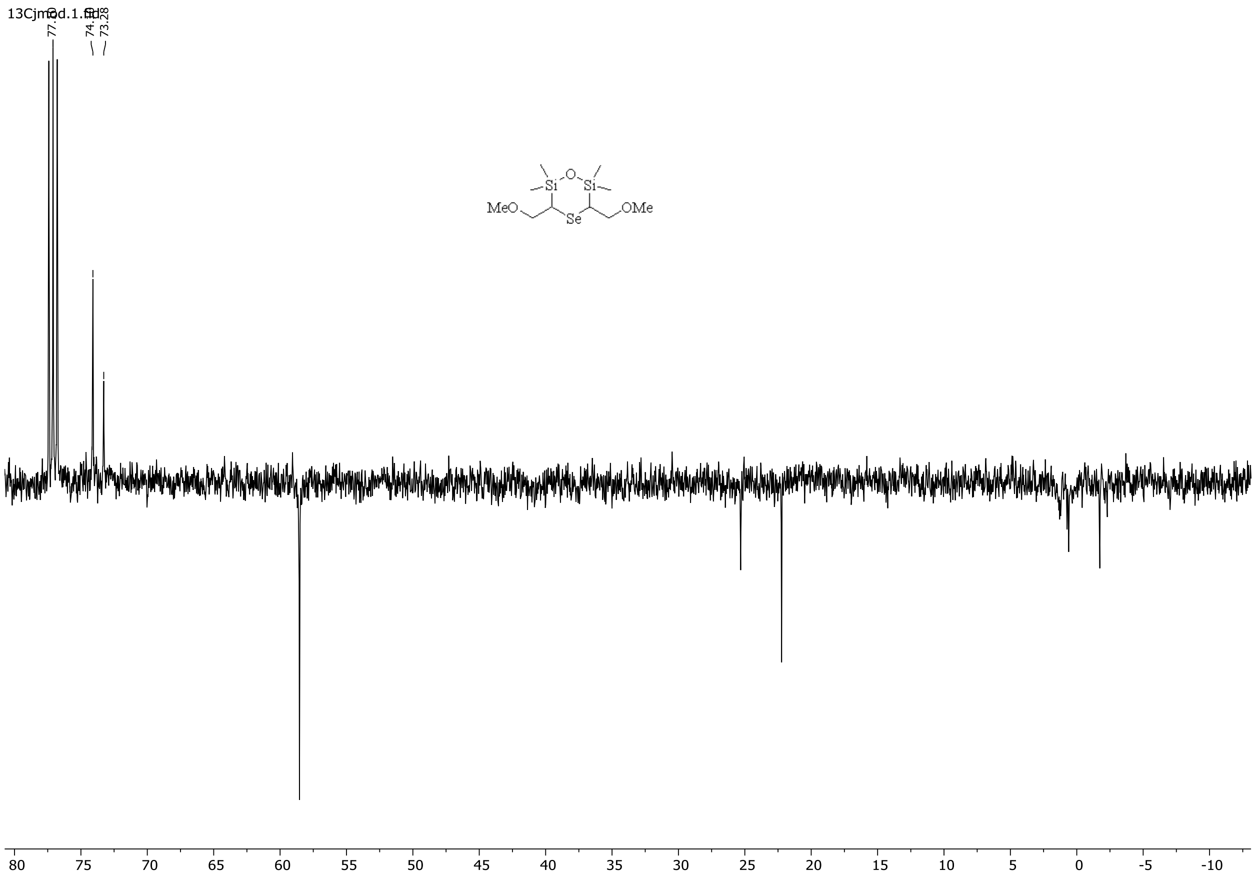

Figure S48. $^{1}J(^{77}Se{-}^{13}C)$ constants of the SeCH signal in ^{13}C NMR spectrum of compound **10**

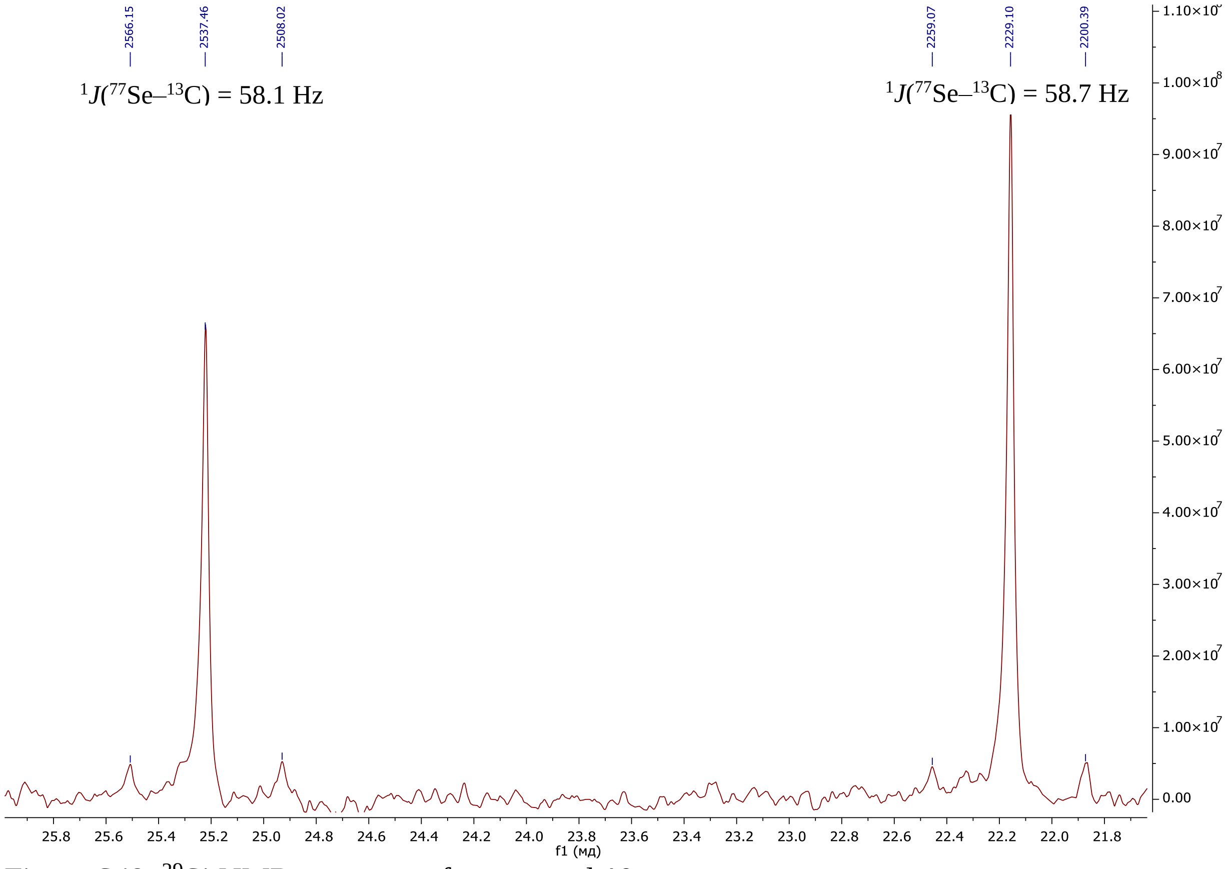

Figure S49. ^{29}Si NMR spectrum of compound **10**

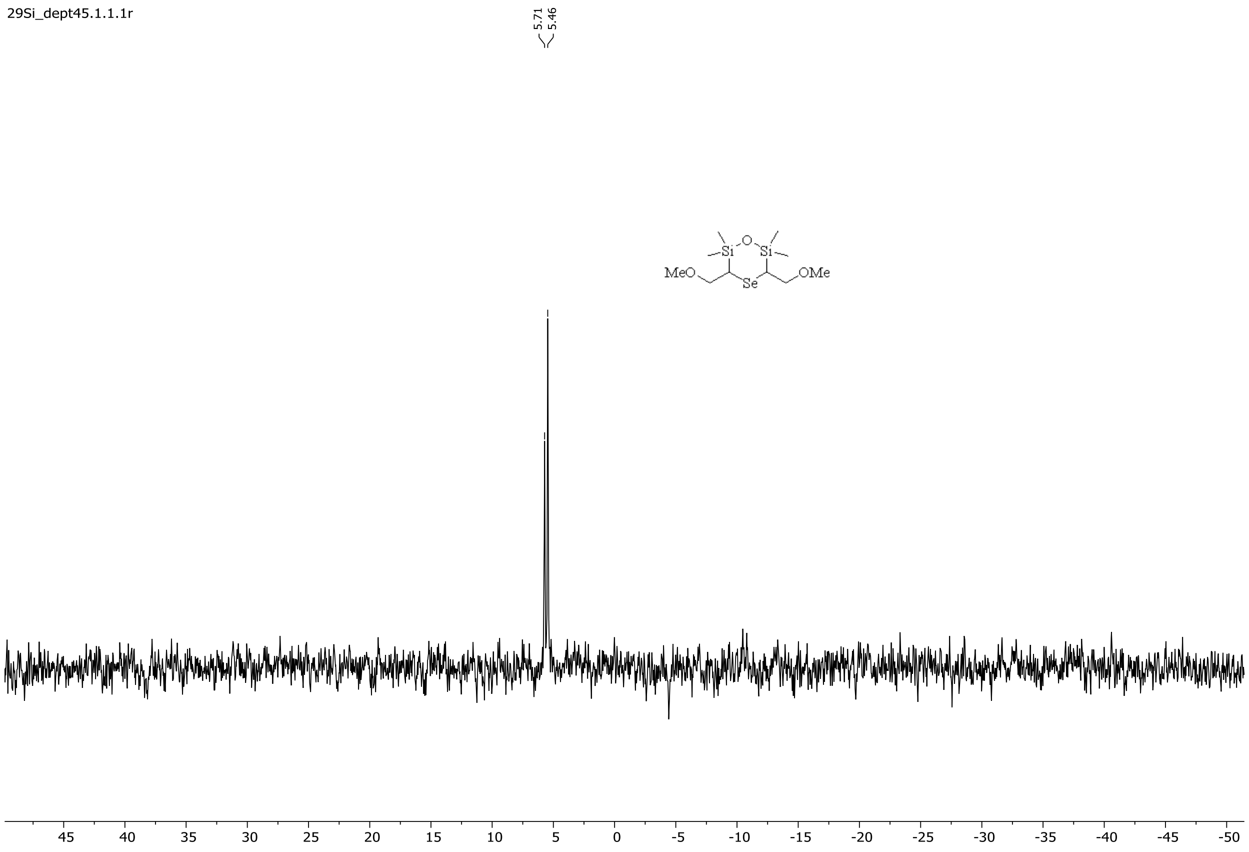

Figure S50. ^{77}Se NMR spectrum of compound **10**

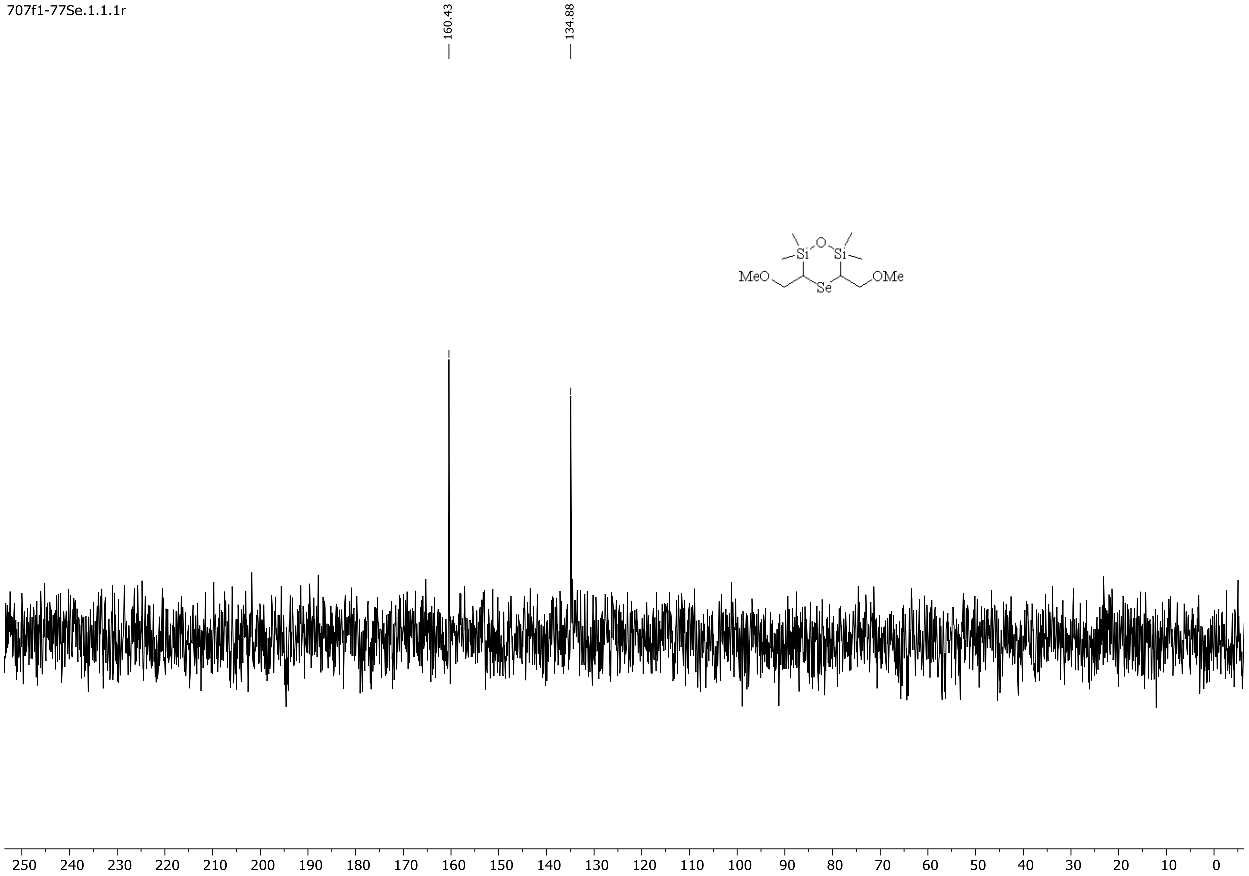

Figure S51. HRMS spectrum of compound **10**

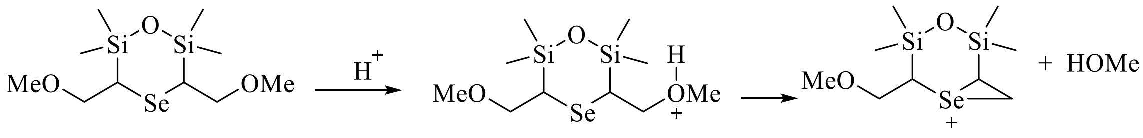
MeO
OMe
Si
O
Si
Se
H+
H
+
+ HOMe

M = 297.0240

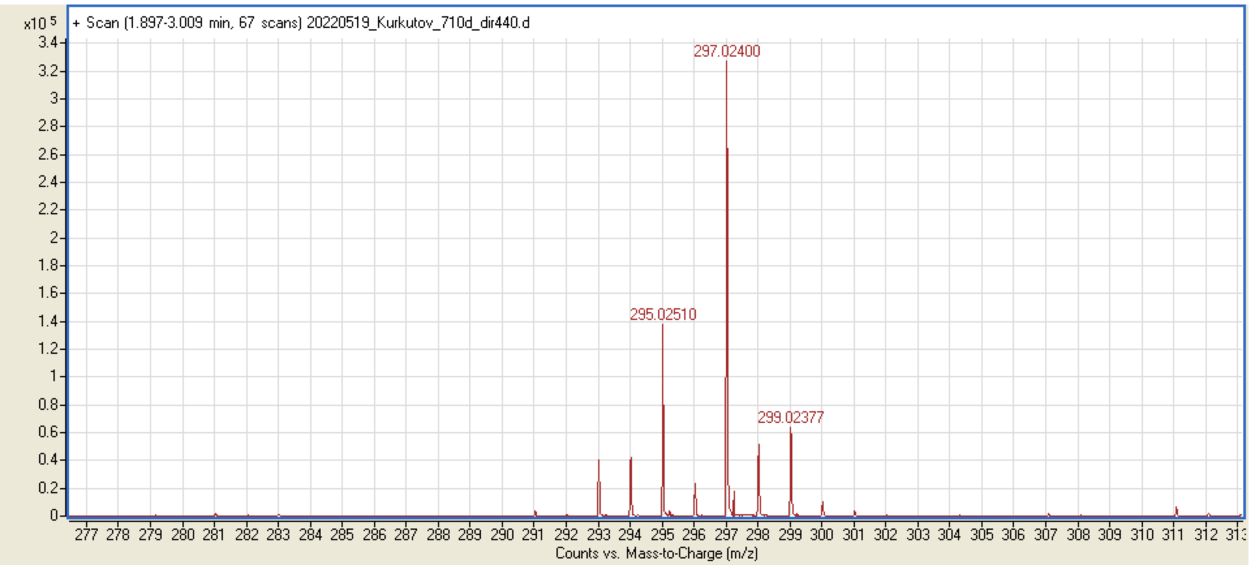
x10 5
+ Scan (1.897-3.009 min, 67 scans) 20220519_Kurkutov_710d_dir440.d
297.02400
295.02510
299.02377
Counts vs. Mass-to-Charge (m/z)
